# Functional correlation tensors in brain white matter and the effects of normal aging

**DOI:** 10.1007/s11682-024-00914-6

**Published:** 2024-09-05

**Authors:** Lyuan Xu, Yurui Gao, Muwei Li, Richard Lawless, Yu Zhao, Kurt G. Schilling, Baxter P. Rogers, Adam W. Anderson, Zhaohua Ding, Bennett A. Landman, John C. Gore

**Affiliations:** 1 Vanderbilt University Institute of Imaging Science, Vanderbilt University Medical Center, 1161 21st Ave. S, Medical Center North, Nashville, TN AA-1105, 37232-2310 USA; 2 Department of Electrical and Computer Engineering, Vanderbilt University, Nashville, TN USA; 3 Department of Biomedical Engineering, Vanderbilt University, Nashville, TN USA; 4 Department of Radiology and Radiological Sciences, Vanderbilt University Medical Center, Nashville, TN USA; 5 Department of Psychiatry and Behavioral Sciences, Vanderbilt University Medical Center, Nashville, TN USA

**Keywords:** Functional correlation tensor, White matter, Resting state, fMRI, BOLD, Aging

## Abstract

Resting state correlations between blood oxygenation level dependent (BOLD) MRI signals from voxels in white matter (WM) are demonstrably anisotropic, so that functional correlation tensors (FCT) may be used to quantify the underlying microstructure of BOLD effects in WM tracts. However, the overall spatial distribution of FCTs and their metrics in specific populations has not yet been established, and the factors that affect their precise arrangements remain unclear. Changes in WM occur with normal aging, and these may be expected to affect FCTs. We hypothesized that FCTs exhibit a characteristic spatial pattern and may show systematic changes with aging or other factors. Here we report our analyses of the FCT characteristics of fMRI images of a large cohort of 461 cognitively normal subjects (190 females, 271 males) sourced from the Open Access Series of Imaging Studies (OASIS), with age distributions of 42 y/o – 95 y/o. Group averages and statistics of FCT indices, including axial functional correlations, radial functional correlations, mean functional correlations and fractional anisotropy, were quantified in WM bundles defined by the JHU ICBM-DTI-81 WM atlas. In addition, their variations with normal aging were examined. The results revealed a dimorphic distribution of changes in FCT metrics with age, with decreases of the functional correlations in some regions and increases in others. Supplementary analysis revealed that females exhibited significant age effects on a greater number of WM areas, but the interaction between age and sex was not significant. The findings demonstrate the reproducibility of the spatial distribution of FCT metrics and reveal subtle regional changes with age.

**Supplementary Information:**

The online version contains supplementary material available at 10.1007/s11682-024-00914-6.

## Introduction

Neuroimaging studies comparing different populations rely on identifying features (such as quantitative measures of structures) whose means and variations are documented such that significant differences may be reliably detected. One approach that serves such goals is the creation of atlases that integrate and average the data from a large number of subjects. Several properties, such as white matter (WM) tracts and functional parcellations, have been condensed into available atlases that thus enable comparisons between different populations or the quantification of the influence of other variables. Here we describe the development of new functional maps that quantify the overall spatial distribution of metrics of functional correlation tensors (FCT) in late adulthood, obtained from resting state studies of brain, and we illustrate the effects of normal aging on FCT characteristics.

Functional magnetic resonance imaging (fMRI) has been widely applied to study gray matter (GM), where changes in blood oxygenation level dependent (BOLD) signals are measured as indirect indicators of neural activity. However, much less work has been focused on corresponding changes in WM, which is irrigated by much reduced blood vasculature (Helenius et al., 2003; Raichle et al., 2001; Rostrup et al., 2000). Recently, considerable evidence has emerged that BOLD signals in WM can also be robustly detected using appropriate methods (Ding et al., 2018; Gawryluk et al., 2014; Gore et al., 2019; Huang et al., 2018; Li et al., 2019). Moreover, WM functional metrics have shown their relevance in monitoring alterations associated with brain disorders (Gao et al., 2020, 2021; Kong et al., 2022) and seasonal variations (Xu et al., 2023). One notable finding is that resting state BOLD signals in WM exhibit local structure-specific anisotropic temporal correlations, and this functional correlational anisotropy within local WM neighborhoods may be characterized by a FCT (Ding et al., 2013, 2016) whose primary orientations often align with local WM structure measured by conventional diffusion tensor imaging. FCTs depend on microvascular, neural and structural factors (Ding et al., 2016), and have previously been used to assess neurological conditions such as pontine strokes (Wang et al., 2019) and mild cognitive impairment (Chen et al., 2017). However, the spatial patterns of FCT metrics within specific populations has not yet been quantified, which is essential for assessing the overall functional architecture in WM. Additionally, as individuals age, the brain undergoes age-related processes that affect both vascular and neural structural levels. Yet, the effects of normal aging on FCTs remains unexplored. We hypothesized that FCTs and their metrics, with a certain spatial distribution, may undergo age-related changes across the normal age span. Despite the existing region-wise studies on age-related variations in long-distance WM FC in aging research (Gao et al., 2023; Li et al., 2023), there is still a deficiency in comprehensive characterization of how aging impacts localized functional synchrony, and such detailed descriptions could enhance our understanding of the functional architecture of WM and its associated aging-related changes. Such insights could be revealed through FCTs by measuring the correlational anisotropy of resting state fMRI signals among neighboring voxels in WM.

The changes with aging of the human brain are of fundamental interest to neuroscience research and have societal implications as the population ages. Brain aging is a significant risk factor for a variety of neurodegenerative conditions, including Alzheimer’s disease (Jockwitz & Caspers, 2021; Koen & Rugg, 2019; Lee & Kim, 2022; Mattson & Arumugam, 2018; Morrison & Hof, 1997). Numerous studies have found that normal aging is accompanied by brain changes at cellular (Lu et al., 2004; Yankner et al., 2008), biochemical (Lodish et al., 2000; Nikhra, 2017), and structural levels (DeCarli et al., 2005; Peters, 2006), which may eventually lead to a decline of cognitive functions, such as memory, attention, and executive functions (Park & Reuter-Lorenz, 2009; Reuter-Lorenz, 2002; Reuter-Lorenz & Park, 2014; Walhovd et al., 2011).

Extensive efforts have been dedicated to imaging studies of changes with brain aging, especially the characterization of the temporal trajectories of changes in brain structure and function (Coupé et al., 2017; Tomasi & Volkow, 2012). Anatomically, reductions in GM volumes have consistently been found in late adulthood (Coupé et al., 2017) and various reports have shown that the thickness of the cerebral cortex decreases with age. Meanwhile, age-related changes of both long-and short-range functional connectivity have been observed in various well-established cortical networks (Tomasi & Volkow, 2012). In addition to age effects on GM, which has been the primary focus of previous studies, mounting evidence has indicated that WM also is affected by brain aging. For example, diffusion tensor imaging (DTI), which allows WM microstructure to be probed non-invasively in vivo (Pierpaoli & Basser, 1996; Pierpaoli et al., 1996), has been used extensively to assess various brain conditions, particularly in the context of the normal aging. By deriving indices from diffusion tensor measurements across different age groups, characteristic trajectories of age effects in fractional anisotropy (FA), axial diffusivity (AD), radial diffusivity (RD), mean diffusivity (MD) and other diffusion indices (Barrick et al., 2010; Bendlin et al., 2010; Cox et al., 2016; Sullivan et al., 2010) have been depicted, demonstrating that WM microstructure is strongly influenced by aging.

Based on these considerations, we conducted analyses of FCTs in WM to establish the overall spatial distribution of FCT metrics among the late adulthood, and then also to evaluate age-related variations in the temporal correlations of BOLD signals. To simplify our approach, we computed indices originating from the eigenvalues associated with FCT’s characteristic directions. These distinctive directions capture the anisotropic correlations between BOLD signals of voxels and the surrounding neighborhoods. Consequently, the eigenvalues linked with these directions serve as indicators of the corresponding functional correlations. Guided by previous index analyses of diffusion tensors, we derived indices of functional tensors based on their eigen parameters. FCT images were first constructed for each subject selected from a cohort of cognitively normal adults spanning a large age range (*N* = 461, age = 42–95 years), from which tensor indices were calculated for each WM voxel. The evolution of FCT indices with age was then assessed at the voxel-level, and with mean FCT indices derived from individual WM tracts (Mori et al., 2008), age effects were then characterized at the bundle-level. Findings from this study provide novel insight into the normal variations and evolution of WM functional structure with age at both micro- and macro- levels.

## Methods

### Data

Data analyzed in this study were obtained from the Open Access Series of Imaging Studies – stage 3 database (LaMontagne et al., 2019) (OASIS-3, https://www.oasis-brains.org), among which 461 subjects (cognitive normal subjects, Clinical Dementia Rating (CDR) = 0) passed quality control processes (see the section ‘Preprocessing’ for the details). There are 190 males and 271 females, whose ages ranged from 42 years to 95 years (age $\:=68.5\pm\:9.1$ years). The Figure S1 presents a histogram regarding the demographic characteristics of all participants. We used the data obtained on their first visits (or screening sessions). The imaging scanners and corresponding protocols are described in detail in a previous report provided by LaMontagne, Pamela J., et al. (LaMontagne et al., 2019). All baseline data of resting state fMRI (rsfMRI) of cognitively normal (CN) subjects and the corresponding T1-weighted (T1w) images were acquired in de-identified form, as well as other demographic information, with institutional review board (IRB) approvals. Briefly, all scans were conducted by the Knight Alzheimer Research Imaging Program at Washington University in St. Louis. MRI was collected on TIM Trio 3T (2 different scanners of this model, labeled as 35177 & 35248), and BioGraph mMR PET-MR 3T (labeled as 51010). The supplementary Table S1 displays the summary of the number of subjects scanned in each site/scanner, along with their corresponding parameters. The Participants were placed in a 20-channel head coil on 3T scanners with foam pad stabilizers placed next to the ears to decrease motion. For the fMRI session, the repetition time (TR) is 2200 ms, the echo time is 27 ms, the voxel size is $\:4\times\:4\times\:4$ mm, and the number of volumes is 164.

### Preprocessing

An automated high-performance pipeline was generated to preprocess the large-scale data (see a previous report (Yan et al., 2016) for more information). Briefly, slice timing was corrected, and head motions were removed from the rsfMRI volumes, and then 24 motion-related parameters and the mean cerebrospinal fluid (CSF) signal were viewed as covariates and regressed out from the BOLD signals. All the procedures were carried out using a customized pipeline based on the DPABI toolbox (Yan et al., 2016). The Computational Anatomy Toolbox (CAT12) was then used to segment GM, WM, and CSF tissues based on the T1w images (Gaser et al., 2022). The fMRI images, along with corresponding tissue masks, were spatially normalized into MNI space (voxel size$\:=2\times\:2\times\:2$ mm^3^) using co-registration and normalizing functions in SPM12 (Penny et al., 2011). The preprocessed results were subjected to a manual quality control procedure in which the passing criteria included: (1) all the preprocessed results must be successfully generated; (2) the maximal translations and rotations of head motion must be less than 2 mm and 2°, respectively; (3) the mean frame-wise displacement (FD) must be less than 0.5 mm; (4) the spatial normalization was acceptable by an expert’s visual inspection. For the manual quality check, processing failures were identified in 12 subjects, and 25 subjects exhibited excessive head motion, leading to a total of 37 subjects failing the quality check step. This left us with 461 subjects for analysis.

### Functional correlational tensor and index analysis

1 $$\:\begin{array}{c}{C}_{ij}={n}_{ij}^{\text{{\rm\:T}}}T{n}_{ij}\end{array}$$

and $\:\varvec{T}$ could be represented as:

2 $$\:\begin{array}{c}T=\left(\begin{array}{ccc}{T}_{11}&\:{T}_{12}&\:{T}_{13}\\\:{T}_{12}&\:{T}_{22}&\:{T}_{23}\\\:{T}_{13}&\:{T}_{23}&\:{T}_{33}\end{array}\right)\end{array}$$

in which the superscript $\:\text{{\rm\:T}}$ represents transpose. It should be noted that the Eq. 1 is a quadratic form in the variables $\:{n}_{x}$, $\:{n}_{y}$, and $\:{n}_{z}$ and it could be rewritten as:

3 $$\eqalign{{C_{ij}} = & {T_{11}}{n_x}^2 + 2{T_{12}}{n_x}{n_y} + {T_{22}}{n_y}^2 \cr & + 2{T_{23}}{n_y}{n_z} + {T_{33}}{n_z}^2 + 2{T_{13}}{n_x}{n_z} \cr}$$

**Fig. 1:**
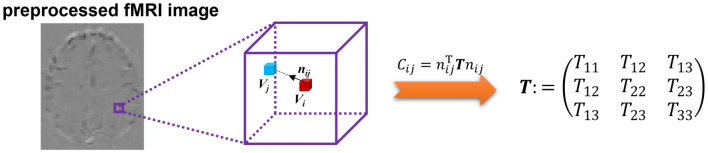
Schematic diagram of the pipeline for calculating the functional correlation tensor. After preprocessing the fMRI image, correlations between each voxel and its neighboring voxels were computed and were fitted into the estimation to achieve the tensor. The purple cube represents the neighborhoods of the given voxel $\:{v}_{i}$

This is a multiple linear regression with the variables $$\:{{n}_{x}}^{2},$$$$\:2{n}_{x}{n}_{y},$$$$\:{{n}_{y}}^{2},$$$$\:2{n}_{y}{n}_{z},$$$$\:{{n}_{z}}^{2},$$$$\:2{n}_{x}{n}_{z}$$ and may be solved using least squares minimization. The resultant coefficients are the components of FCT (i.e., $$\:{T}_{11},$$$$\:{T}_{12},$$$$\:{T}_{13}$$, $$\:{T}_{22},$$$$\:{T}_{23},$$$$\:{T}_{33}$$). In this work, we used a $$\:7\times\:7\times\:7$$ neighborhood to estimate the tensor and the squares of Pearson’s correlation coefficients of preprocessing fMRI signals between voxels as the degree of temporal correlation. The data analysis pipeline is shown in Fig. 1.

By analogy to the use of diffusion quantities AD, RD, MD and FA in DTI, we calculate three metrics of FCT that quantifies the degree of anisotropic correlations. Suppose the eigenvalues of a FCT are $\:{\lambda\:}_{1}$, $\:{\lambda\:}_{2}$, $\:{\lambda\:}_{3}$, and $\:{\lambda\:}_{1}\ge\:{\lambda\:}_{2}\ge\:{\lambda\:}_{3}.$ We can define:

axial functional correlation (axial FC):

4 $$\:\begin{array}{c}axial\:FC={\lambda\:}_{1}\end{array}$$

radial functional correlation (radial FC):

5 $$\:\begin{array}{c}radial\:FC=\:\frac{1}{2}\left({\lambda\:}_{2}+{\lambda\:}_{3}\right)\end{array}$$

and fractional anisotropy of FCT (FA_FCT, ‘FCT’ is suffixed to distinguish it from FA in DTI):

6 $$\:\begin{array}{c}FA=\sqrt{\frac{3}{2}}\frac{\sqrt{{\left({\lambda\:}_{1}-\widehat{\lambda\:}\right)}^{2}+{\left({\lambda\:}_{2}-\widehat{\lambda\:}\right)}^{2}+{\left({\lambda\:}_{3}-\widehat{\lambda\:}\right)}^{2}}}{\sqrt{{\lambda\:}_{1}^{2}+{\lambda\:}_{2}^{2}+{\lambda\:}_{3}^{2}}}\end{array}$$

where mean FC could be defined as:

7 $$\:\begin{array}{c}mean\:FC=\widehat{\lambda\:}=\frac{1}{3}\left({\lambda\:}_{1}+{\lambda\:}_{2}+{\lambda\:}_{3}\right)\end{array}$$

**Fig. 2:**
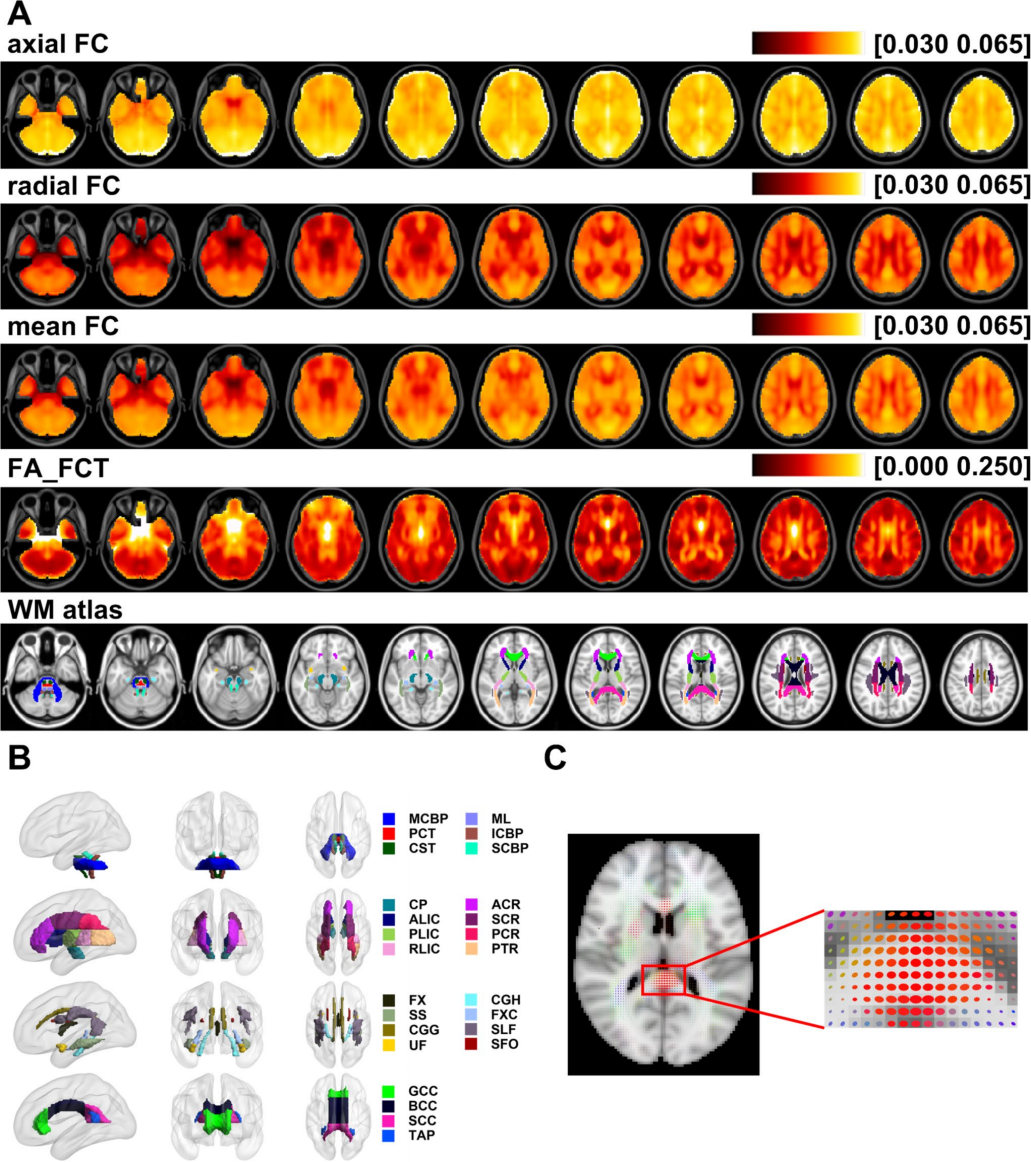
(**A**) Averaged adjusted maps of axial FC, radial FC, mean FC, FA_FCT across all subjects and JHU ICBM-DTI-81 WM atlas used in this work. (**B**) WM bundles defined by JHU ICBM-DTI-81 WM atlas. The abbreviations in the right column are: (1) Tracts in the Brainstem: MCBP: middle cerebellar peduncle; ML: medial lemniscus; PCT: pontine crossing tract; ICBP: inferior cerebellar peduncle; CST: corticospinal tract; SCBP: superior cerebellar peduncle; (2) Projection fibers: CP: cerebral peduncle; ALIC: anterior limb of internal capsule; PLIC: posterior limb of internal capsule; RLIC: retrolenticular part of the internal capsule; ACR: anterior corona radiata; SCR: superior corona radiata; PCR: posterior corona radiata; PTR: posterior thalamic radiation; (3) Association fibers: FX: fornix; SS: sagittal stratum; CGG: cingulum in the cingulate gyrus; CGH: cingulum in the hippocampus; FXC: fornix (cres); SLF: superior longitudinal fasciculus; SFO: superior fronto-occipital fasciculus; UF: uncinate fasciculus; (4) Commissural fibers: GCC: genu of corpus callosum; BCC: body of corpus callosum; SCC: splenium of corpus callosum; TAP: tapetum. (**C**) FCT ellipsoid map of a subject weighted by its own FCT FA and localized magnification image of the corpus callosum region

After the FCT of each voxel was derived for each subject, three indices of FCT were calculated at voxel-level and tract-level for statistical analyses of age effects. In this research, we exclusively focus on examining the age-related effects on FCT indices within the deep WM, as defined by the JHU ICBM-DTI-81 WM atlas (Mori et al., 2008). For the tract-level, indices were also averaged for voxels of each WM tract defined by the JHU ICBM-DTI-81 WM atlas (Mori et al., 2008). The WM tracts defined by the atlas and their corresponding partition are as follow (as shown in Fig. 2): (1) Tracts in the Brainstem: MCBP: middle cerebellar peduncle; ML: medial lemniscus; PCT: pontine crossing tract; ICBP: inferior cerebellar peduncle; CST: corticospinal tract; SCBP: superior cerebellar peduncle; (2) Projection fibers: CP: cerebral peduncle; ALIC: anterior limb of internal capsule; PLIC: posterior limb of internal capsule; RLIC: retrolenticular part of the internal capsule; ACR: anterior corona radiata; SCR: superior corona radiata; PCR: posterior corona radiata; PTR: posterior thalamic radiation; (3) Association fibers: FX: fornix; SS: sagittal stratum; CGG: cingulum in the cingulate gyrus; CGH: cingulum in the hippocampus; FXC: fornix (cres); SLF: superior longitudinal fasciculus; SFO: superior fronto-occipital fasciculus; UF: uncinate fasciculus; (4) Commissural fibers: GCC: genu of corpus callosum; BCC: body of corpus callosum; SCC: splenium of corpus callosum; TAP: tapetum.

### Regional homogeneity (ReHo)

Regional homogeneity (ReHo) value was generated following the previously mentioned pipeline based on the DPABI toolbox (Yan et al., 2016). The calculation of ReHo adopts the method proposed by Zang et al. (Zang et al., 2004). In a nutshell, Kendall’s coefficient of concordance (KCC) was employed to assess the similarity between the time series of a specific voxel and its nearest neighbors, adopting a voxel-wise approach. After ReHo map was generated for each subject, ReHo was also averaged for voxels of each WM tract defined by JHU ICBM-DTI-81 WM atlas (Mori et al., 2008).

### Statistical analysis

To quantify age effects on FCTs, we conducted multiple linear regression to model the measurements over the span of adulthood. A linear effects model was adopted with head motion parameters (the mean Power’s FD (Power et al., 2012), sex and sites as other covariates:

8 $$\eqalign{ y = & {\beta _c} + \beta {x_{age}} + {\beta _{sex}}{x_{sex}} \cr & + {\beta _{FD}}{x_{FD}} + {\beta _{site}}{x_{site}} + \varepsilon \cr}$$

in which y represents the FCT metrics to be analyzed (all the metrics were standardized using z-scores before being incorporated into the regression model) and $$\:{\beta\:}_{c},$$$$\:\beta,\:$$$$\:{\beta\:}_{sex},$$$$\:{\beta\:}_{FD}$$ and $$\:{\beta\:}_{site}$$ are the corresponding coefficients of the covariables (note that $$\:{x}_{sex}$$ and $$\:{x}_{site}$$ was a categorical variable related to the sex of subjects). To determine the significance of age effects, $$\:p$$ values for the t-statistic of the hypothesis test that the coefficients $$\:\beta\:$$ are equal to zero or not were calculated and then were adjusted for multiple comparisons using false discovery rate (FDR) with the method of Benjamini and Hochberg (Benjamini & Hochberg, 1995), generating q-values. It was concluded that the metric was significantly affected by age if the corresponding q-values are less than 0.05 ($$\:q<0.05$$). The value $$\:\beta\:$$ was taken as the association parameter of the age effects, and $$\:\beta\:>0$$ means there was a positive linear trend with age, and $$\:\beta\:<0$$ led to a negative linear trend with increasing age. We initially performed voxel-wise age effect analyses for each FCT metric, generating corresponding coefficient distribution maps ($$\:q<0.05$$, FDR-corrected) within the WM. Subsequently, we conducted age effect analyses for each WM-averaged FCT metric and produced corresponding estimation and scatter plots. In these plots, the vertical axis represents corresponding values with unrelated variables (sex, site, and head motion parameters) regressed out. Furthermore, we expand our research by analyzing the age effects on FCT parameters within the female or male groups, and the corresponding equation 8 is extended as follows:

9 $$\eqalign{ y = &\, {\beta _c} + \beta {x_{age}} + {\beta _{FD}}{x_{FD}} \cr & + {\beta _{site}}{x_{site}} + \varepsilon \cr}$$

Basically, we excluded the sex variable, and conducted separate characterization of age effects on FCT indices for female and male groups. The linear coefficients related to the respective age effects on WM-averaged FCT indices for different WM tracts are presented in the supplementary Table S2.

## Results

A total number of 461 cognitive normal subjects (42–95 years, mean age = $\:68.5\pm\:9.1\:$years) were obtained through OASIS-3 database. For each subject, FCT was calculated, and the corresponding FCT indices, axial FC, radial FC, and FA_FCT, were also generated. Averaged maps of axial, radial FC, mean FC and FA_FCT across all subjects, along with the WM atlas, were shown in Fig. 2. Additionally, FCT ellipsoid map of a subject weighted by its own FCT FA and localized magnification image of the corpus callosum region was also shown in Fig. 2. First, we analyzed age-related variations of each FCT index at voxel level. Additionally, FCT indices were averaged for WM tracts defined by the JHU ICBM-DTI-81 WM atlas (Mori et al., 2008) (as shown in Fig. 2), and coefficients of age effects of averaged FCT indices were calculated.

### Age effects on axial functional correlation

**Fig. 3:**
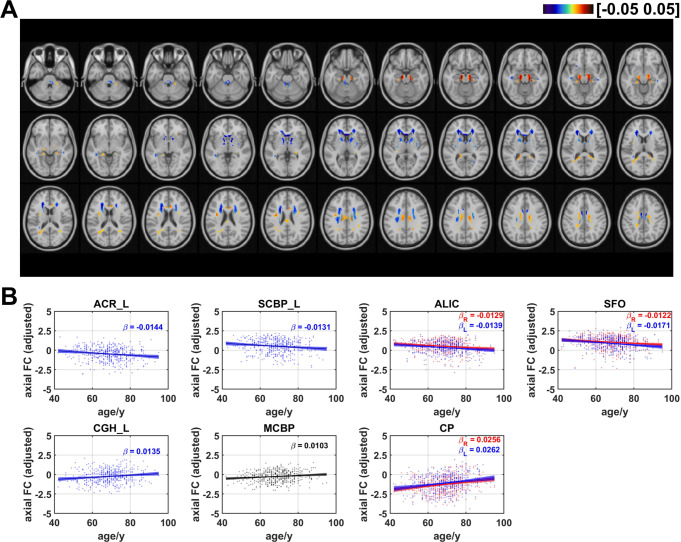
Age-related coefficients of axial functional correlation (axial FC). (**A**) Voxel-wise map of linear age effects on adjusted axial FC. All p values for the t-statistic test have been FDR corrected using Benjamini and Hochberg’s method. Only the voxels that have passed significance test were plotted. (**B**) Plots of significant age-related relationships between adjusted axial FC and age for different WM tracts. Age effects of axial FC of WM tracts in left and right hemisphere are plotted in blue and red respectively. Same as the voxel-wise analysis, all p-values for t-statistic test have been corrected and q-values were generated

Figure 3 showed the distribution of coefficients of age effects on axial FC. Negative age effects were discovered in most regions of WM, including areas in cingulum in the cingulate gyrus (CGG), superior fronto-occipital fasciculus (SFO), genu of corpus callosum (GCC), anterior and superior part of corona radiata (ACR and SCR), anterior limb of internal capsule (ALIC), and superior cerebellar peduncle (SCBP). The results also revealed that some voxels in several regions demonstrated positive linear age effects on axial FC, including the body of corpus callosum (BCC), superior longitudinal fasciculus (SLF), cingulum in hippocampus (CGH), cerebral peduncle (CP), and middle cerebellar peduncle (MCBP). The ratios of the number of voxels in WM that revealed negative and positive linear age effects on axial FC are respectively 55.7% and 44.3% (as shown in Fig. 7). Axial FCs were then averaged for each WM bundle and the results showed that age effects on mean axial FC revealed a negative trend in left ACR ($\:\beta\:=-0.0144$, $\:q<0.05$), bilateral ALIC (left: $\:\beta\:=-0.0139$, $\:q<0.01$; right: $\:\beta\:=-0.0129$, $\:q<0.05$), left SCBP ($\:\beta\:=-0.0131$, $\:q<0.05$) and bilateral SFO (left: $\:\beta\:=-0.0171$, $\:q<0.01$; right: $\:\beta\:=-0.0122$, $\:q<0.05,$) while positive linear age effects were found in bilateral CP (left: $\:\beta\:=0.0262$, $\:q<0.001$; right: $\:\beta\:=0.0256$, $\:q<0.001,$) left CGH ($\:\beta\:=0.0135$, $\:q<0.05$) and MCBP ($\:\beta\:=0.0103$, $\:q<0.05$). The results revealed that that there are more WM tracts showing a negative age-related correlation compared to positive correlations, with the majority of them being projection fibers, as shown in Figure S2. Table 1 summarizes the age effects of axial FC (as well as radial FC, mean FC and FA_FCT) and the corresponding $\:q$ values of all WM tracts.

**Table 1:** Coefficients $\:\beta\:$ and $\:q$ values of linear age-effects of axial FC, radial FC, mean FC, and FA_FCT averaged for WM tracts, in which significant results are highlighted in bold (significant level = 0.05)

WM tract		Axial FC		Radial FC		Mean FC		FA_FCT		WM tract description
		$\:\beta\:$	$\:q$	$\:\beta\:$	$\:q$	$\:\beta\:$	$\:q$	$\:\beta\:$	$\:q$	
MCBP	-	**0.0103**	**0.0364**	0.0060	0.2077	0.0074	0.1141	0.0013	0.7700	middle cerebellar peduncle
PCT	-	-0.0072	0.2867	**-0.0159**	**0.0478**	-0.0147	0.0593	**0.0194**	**0.0089**	pontine crossing tract
GCC	-	-0.0086	0.1592	-0.0019	0.7858	-0.0038	0.5521	-0.0043	0.4880	genu of corpus callosum
BCC	-	0.0113	0.0638	0.0040	0.5125	0.0059	0.3324	-0.0025	0.6174	body of corpus callosum
SCC	-	0.0066	0.1592	**0.0173**	**0.0002**	**0.0153**	**0.0017**	**-0.0256**	**0.0000**	splenium of corpus callosum
FX	-	-0.0039	0.4699	**-0.0199**	**0.0048**	**-0.0173**	**0.0086**	**0.0205**	**0.0077**	fornix
CST	R	0.0097	0.1841	-0.0006	0.9294	0.0017	0.8776	0.0076	0.2627	corticospinal tract
	L	0.0121	0.1077	-0.0022	0.7858	0.0009	0.9111	0.0107	0.0969	
ML	R	-0.0121	0.1428	-0.0154	0.0507	-0.0149	0.0593	0.0149	0.0502	medial lemniscus
	L	-0.0070	0.3672	-0.0125	0.1166	-0.0114	0.1278	**0.0168**	**0.0188**	
ICBP	R	0.0042	0.5213	0.0048	0.4991	0.0049	0.4454	-0.0046	0.3879	inferior cerebellar peduncle
	L	0.0104	0.1072	0.0092	0.1418	0.0098	0.1131	-0.0070	0.2185	
SCBP	R	-0.0089	0.1602	**-0.0201**	**0.0026**	**-0.0182**	**0.0072**	**0.0222**	**0.0004**	superior cerebellar peduncle
	L	**-0.0131**	**0.0364**	**-0.0215**	**0.0007**	**-0.0203**	**0.0020**	**0.0234**	**0.0001**	
CP	R	**0.0256**	**0.0005**	0.0094	0.2045	0.0135	0.0602	0.0047	0.5419	cerebral peduncle
	L	**0.0262**	**0.0005**	0.0082	0.2916	0.0127	0.0872	0.0061	0.4499	
ALIC	R	**-0.0129**	**0.0228**	**-0.0159**	**0.0037**	**-0.0159**	**0.0043**	**0.0139**	**0.0056**	anterior limb of internal capsule
	L	**-0.0139**	**0.0076**	**-0.0129**	**0.0138**	**-0.0135**	**0.0112**	**0.0101**	**0.0430**	
PLIC	R	-0.0048	0.3014	**-0.0108**	**0.0244**	-0.0098	0.0528	**0.0105**	**0.0156**	posterior limb of internal capsule
	L	-0.0055	0.2205	**-0.0119**	**0.0138**	**-0.0106**	**0.0328**	**0.0131**	**0.0026**	
RLIC	R	0.0027	0.5601	0.0090	0.0728	0.0080	0.1075	**-0.0137**	**0.0034**	retrolenticular part of the internal capsule
	L	0.0015	0.7355	0.0053	0.3064	0.0046	0.3755	-0.0088	0.0738	
ACR	R	-0.0107	0.0718	-0.0004	0.9294	-0.0029	0.6398	-0.0060	0.2088	anterior corona radiata
	L	**-0.0144**	**0.0221**	-0.0015	0.7897	-0.0045	0.4454	-0.0043	0.3517	
SCR	R	-0.0013	0.7418	0.0063	0.2077	0.0046	0.3648	-0.0075	0.0823	superior corona radiata
	L	-0.0036	0.4207	0.0047	0.3064	0.0030	0.5222	-0.0051	0.1876	
PCR	R	0.0049	0.3672	**0.0174**	**0.0002**	**0.0152**	**0.0020**	**-0.0198**	**0.0000**	posterior corona radiata
	L	0.0026	0.6046	**0.0127**	**0.0104**	**0.0108**	**0.0402**	**-0.0139**	**0.0008**	
PTR	R	0.0013	0.7723	**0.0149**	**0.0057**	**0.0122**	**0.0312**	**-0.0239**	**0.0000**	posterior thalamic radiation
	L	0.0033	0.5433	**0.0241**	**0.0000**	**0.0203**	**0.0005**	**-0.0334**	**0.0000**	
SS	R	-0.0059	0.2823	0.0030	0.6040	0.0009	0.9111	-0.0073	0.1616	sagittal stratum
	L	-0.0097	0.0638	0.0040	0.5158	0.0009	0.9111	-0.0091	0.0968	
CGG	R	-0.0065	0.1757	-0.0039	0.4793	-0.0046	0.3648	-0.0006	0.8882	cingulum in the cingulate gyrus
	L	-0.0073	0.1592	-0.0028	0.5690	-0.0039	0.4454	-0.0022	0.6174	
CGH	R	0.0114	0.0638	0.0034	0.5665	0.0055	0.3690	0.0095	0.0823	cingulum in the hippocampus
	L	**0.0135**	**0.0228**	0.0086	0.1340	0.0100	0.0825	-0.0013	0.8244	
FXC	R	0.0050	0.3505	0.0033	0.5498	0.0040	0.4454	0.0000	0.9906	fornix (cres)
	L	0.0028	0.5433	-0.0008	0.8835	0.0000	1.0000	0.0023	0.6174	
SLF	R	0.0066	0.1718	**0.0096**	**0.0478**	0.0091	0.0593	**-0.0115**	**0.0056**	superior longitudinal fasciculus
	L	0.0040	0.4081	0.0091	0.0582	0.0080	0.0913	**-0.0109**	**0.0103**	
SFO	R	**-0.0122**	**0.0364**	-0.0084	0.1340	-0.0098	0.0825	0.0086	0.0968	superior fronto-occipital fasciculus
	L	**-0.0171**	**0.0013**	-0.0084	0.1340	-0.0107	0.0593	0.0058	0.2812	
UF	R	0.0099	0.2081	-0.0022	0.7897	0.0006	0.9467	0.0119	0.1317	uncinate fasciculus
	L	0.0105	0.1841	-0.0046	0.5665	-0.0010	0.9111	0.0136	0.0510	
TAP	R	-0.0007	0.8899	0.0115	0.0796	0.0092	0.1455	**-0.0227**	**0.0002**	tapetum
	L	-0.0040	0.5536	0.0049	0.5125	0.0033	0.6398	**-0.0144**	**0.0167**	

### Age effects on radial functional correlation

**Fig. 4:**
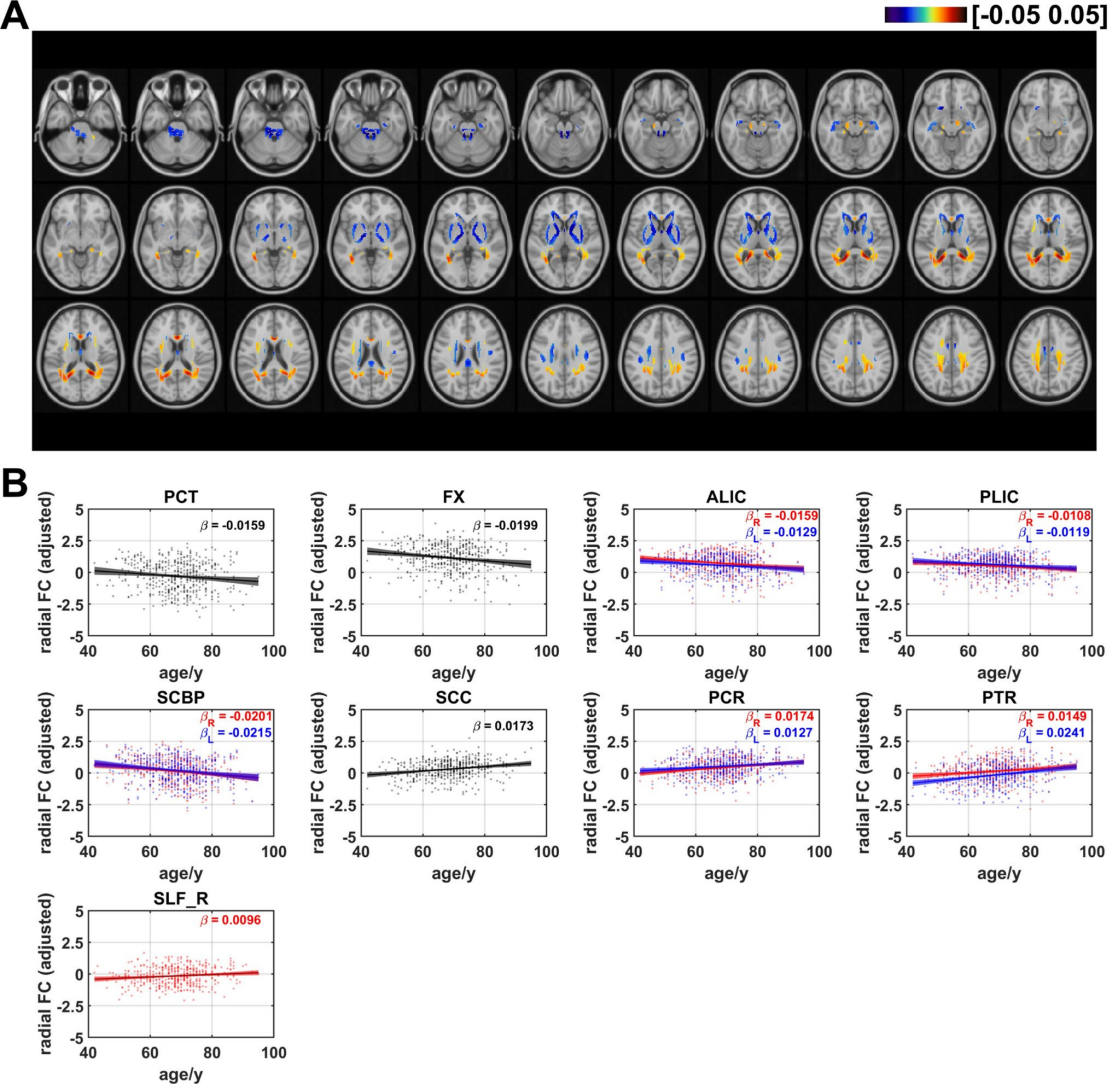
Age-related coefficients of radial functional correlation (radial FC). (**A**) Voxel-wise map of linear age effects on adjusted radial FC. All p values for the t-statistic test have been FDR corrected using Benjamini and Hochberg’s method. Only the voxels that passed significance test were plotted. (**B**) Plots of significant age-related relationships between adjusted radial FC and age for different WM tracts. Age effects of radial FC of WM tracts in left and right hemisphere are plotted in blue and red respectively. Same as the voxel-wise analysis, all p-values for t-statistic test have been corrected and q-values were generated

The age effects on radial FC are shown in Fig. 4. Negative linear age effects showed up in areas in CGG, fornix (FX), GCC, BCC, SCR, ALIC, posterior limb of internal capsule (PLIC), SCBP, pontine crossing tracts (PCT), MCBP. In comparison with the negative age effects, positive linear trends with increasing age occurred in areas in posterior corona radiata (PCR), SLF, splenium of corpus callosum (SCC), posterior thalamic radiation (PTR). The proportion of the number of voxels with negative age effects decreased for radial FC. The ratios of the number of voxels in WM that revealed negative and positive linear age effects on radial FC are respectively 49.9% and 50.1% (as shown in Fig. 7). For the averaged radial FC of WM bundles, the results revealed negative age effects in bilateral ALIC (left: $$\:\beta\:=-0.0129$$, $$\:q<0.05$$; right: $$\:\beta\:=-0.0159$$, $$\:q<0.01,$$) FX ($$\:\beta\:=-0.0199$$, $$\:q<0.01$$), PCT ($$\:\beta\:=-0.0159$$, $$\:q<0.05,$$) bilateral PLIC (left: $$\:\beta\:=-0.0119,$$$$\:q<0.05$$; right: $$\:\beta\:=-0.0108$$, $$\:q<0.05$$) and bilateral SCBP (left: $$\:\beta\:=-0.0215$$, $$\:q<0.001$$; right: $$\:\beta\:=-0.0201$$, $$\:q<0.01$$). Positive linear age effects were detected in bilateral PCR (left: $$\:\beta\:=0.0104$$, $$\:q<0.05$$; right: $$\:\beta\:=0.0174$$, $$\:q<0.001,$$) bilateral PTR (left: $$\:\beta\:=0.0241$$, $$\:q<0.0001$$; right: $$\:\beta\:=0.0149$$, $$\:q<0.01$$), SCC ($$\:\beta\:=0.0173$$, $$\:q<0.001$$) and right SLF ($$\:\beta\:=0.0096$$, $$\:q<0.05$$). The results demonstrated that the majority of WM tracts exhibiting negative age-related effects are located in projection fibers and brainstem. Among the WM tracts with positive age effects on radial FC, the majority belonged to projection fibers, along with one tract in association fiber (right SLF), and one fiber in commissural fibers (SCC), as shown in Figure S3.

### Age effects on mean functional correlation

**Fig. 5:**
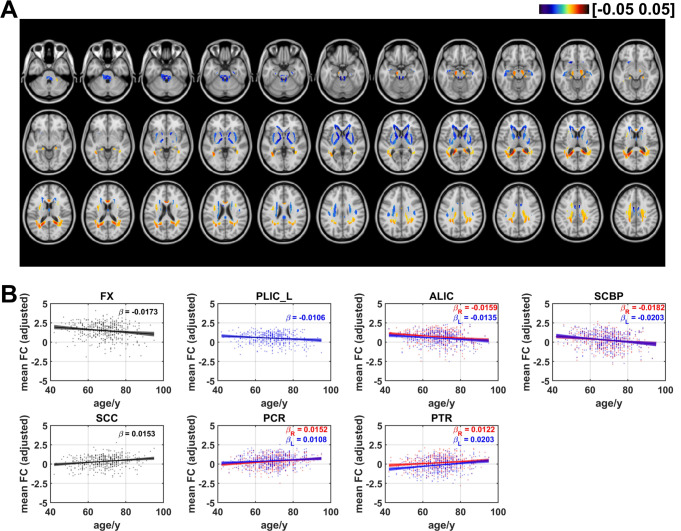
Age-related coefficients of mean functional correlation (mean FC). (**A**) Voxel-wise map of linear age effects on adjusted mean FC. All p values for the t-statistic test have been FDR corrected using Benjamini and Hochberg’s method. Only the voxels that passed significance test were plotted. (**B**) Plots of significant age-related relationships between adjusted mean FC and age for different WM tracts. Age effects of mean FC of WM tracts in left and right hemisphere are plotted in blue and red respectively. Same as the voxel-wise analysis, all p-values for t-statistic test have been corrected and q-values were generated

The age effects on mean FC are shown in Fig. 5. Negative linear age effects showed up in areas in CGG, FX, GCC, BCC, SCR, ALIC, PLIC, SCBP, PCT and MCBP. In comparison with the negative age effects, positive linear trends with increasing age occurred in areas in PCR, SLF, SCC, and PTR. The ratios of the number of voxels in WM that revealed negative and positive linear age effects on mean FC are respectively 50.9% and 49.1% (as shown in Fig. 7). For the averaged mean FC of WM bundles, the results revealed bilateral ALIC (left: $\:\beta\:=-0.0135$, $\:q<0.05$; right: $\:\beta\:=-0.0159$, $\:q<0.01$), FX ($\:\beta\:=-0.0173$, $\:q<0.01$), left PLIC ($\:\beta\:=-0.0106$, $\:q<0.05$) and bilateral SCBP (left: $\:\beta\:=-0.0203$, $\:q<0.01$; right: $\:\beta\:=-0.0182$, $\:q<0.01$). Positive linear age effects were detected in bilateral PCR (left: $\:\beta\:=0.0108$, $\:q<0.05$; right: $\:\beta\:=0.0152$, $\:q<0.01,$) bilateral PTR (left: $\:\beta\:=0.0203$, $\:q<0.001$; right: $\:\beta\:=0.0122$, $\:q<0.05$) and SCC ($\:\beta\:=0.0153$, $\:q<0.01$). The distribution of the age effect on mean FC was similar to that on radial FC. WM tracts in brainstem, projection fibers and one WM tract in association fibers demonstrated negative linear age effects on mean FC, while four tracts in projection fibers and one fiber in commissural fibers (SCC) revealed positive age effects, as shown in Figure S4.

### Age effects on fractional anisotropy of FCT

**Fig. 6:**
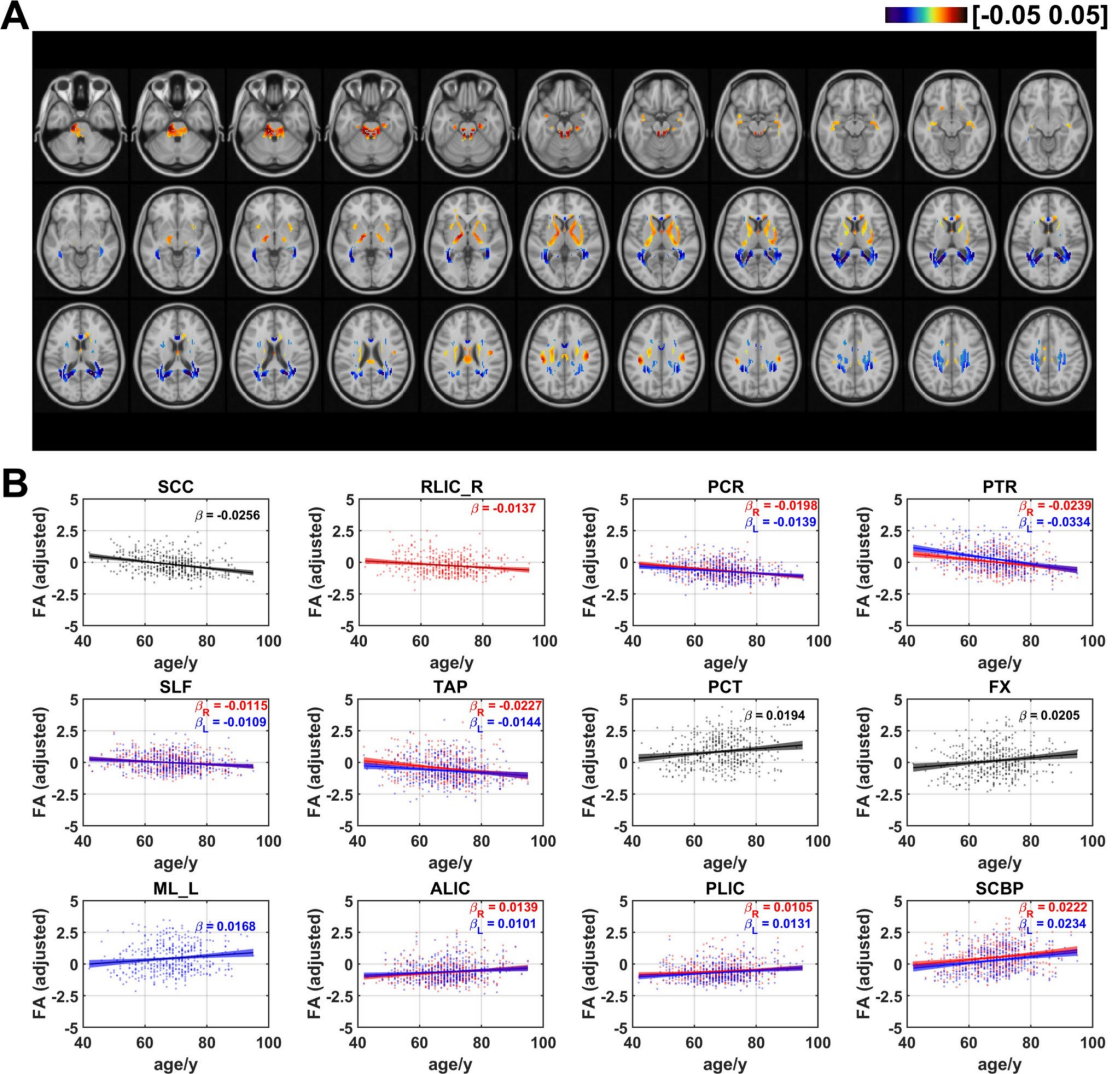
Age-related coefficients of FA of FCT. (**A**) Voxel-wise map of linear age effects on adjusted FA of FCT. All p values for the t-statistic test have been FDR corrected using Benjamini and Hochberg’s method. Only the voxels that have passed significance test were plotted. (**B**) Plots of significant age-related relationships between adjusted FA of FCT and age for different WM tracts. Age effects of FA_FCT of WM tracts in left and right hemisphere are plotted in blue and red respectively. Same as the voxel-wise analysis, all p-values for t-statistic test have been corrected and q-values were generated

**Fig. 7:**
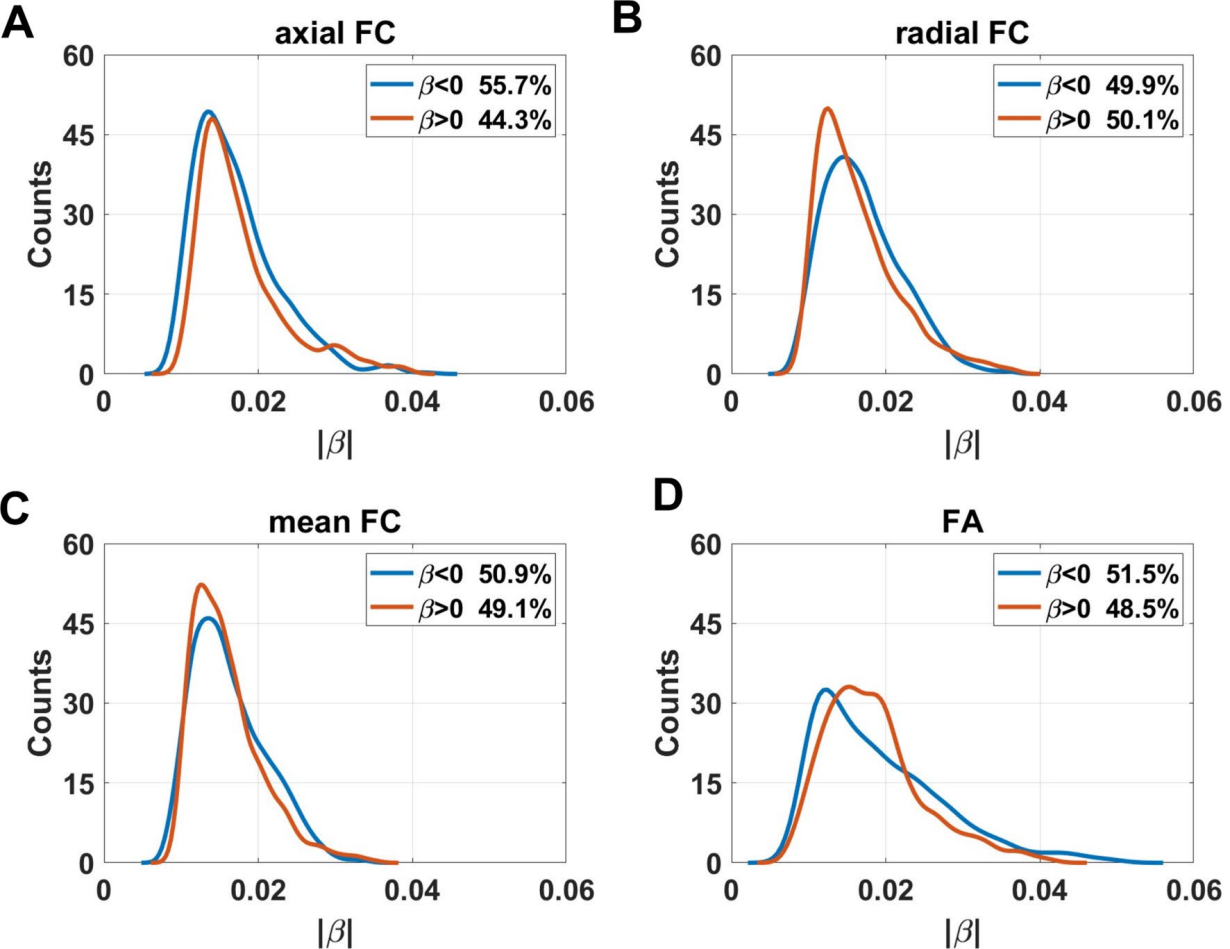
Distribution of linear coefficients $\:\beta\:$ of age-effects of (**A**) axial FC, (**B**) radial FC, (**C**) mean FC and (**D**) FA of FCT

The FA_FCT distributions in WM are shown in Fig. 6. Negative linear age effects occurred in PCR, SLF, retrolenticular part of internal capsule (RLIC), SCC, tapetum (TAP), and PTR. On the other hand, positive age effects occurred in BCC, ALIC, PLIC, FX, medial lemniscus (ML), PCT, SCBP. The ratios of voxels in WM that demonstrated negative and positive linear age effects on FA_FCT are respectively 51.5% and 48.5% (as shown in Fig. 7). In terms of age effects of FA_FCT on WM bundles, negative effects occurred in bilateral PCR (left: $$\:\beta\:=-0.0139$$, $$\:q<0.001;$$ right: $$\:\beta\:=-0.0198$$, $$\:q<0.0001$$), bilateral PTR (left: $$\:\beta\:=-0.0334$$, $$\:q<0.0001;$$ right: $$\:\beta\:=-0.0239$$, $$\:q<0.0001,$$) right RLIC ($$\:\beta\:=-0.0137$$, $$\:q<0.01$$), SCC ($$\:\beta\:=-0.0256$$, $$\:q<0.0001$$), bilateral SLF (left: $$\:\beta\:=-0.0109$$, $$\:q<0.05;$$ right: $$\:\beta\:=-0.0115$$, $$\:q<0.01$$) and bilateral TAP (left: $$\:\beta\:=-0.0144$$, $$\:q<0.05$$; right: $$\:\beta\:=-0.0227$$, $$\:q<0.001)$$. Additionally, the results revealed that positive effects showed up in bilateral ALIC (left: $$\:\beta\:=0.0101;$$, $$\:q<0.05$$ right: $$\:\beta\:=0.0139,$$$$\:q<0.01),$$ FX ($$\:\beta\:=0.0205$$, $$\:q<0.01),$$ left ML ($$\:\beta\:=0.0168,$$$$\:q<0.05),$$ PCT ($$\:\beta\:=0.0194,$$$$\:q<0.01)$$ bilateral PLIC (left: $$\:\beta\:=0.0131,$$$$\:q<0.01;$$ right: $$\:\beta\:=0.0105$$, $$\:q<0.05$$) and bilateral SCBP (left: $$\:\beta\:=0.0234$$, $$\:q<0.001$$; right: $$\:\beta\:=0.0222$$, $$\:q<0.001$$). It could be shown that the distribution pattern of age effects on WM FA_FCT was roughly opposite to that of radial FC. FA_FCT exhibited positive age effects in the regions which correspond to the area that revealed negative age effects in radial FC (PCT, SCBP, ALIC and PLIC), while other WM tracts in projection fibers, association fibers and commissural fibers revealed negative age effects, as shown in Figure S5. Furthermore, it is particularly noteworthy that, in comparison to the age effects observed in ReHo (as shown in Figure S6), the WM tracts in the brainstem that showed significant age effects are all associated with a positive age-related increase, as also observed in ReHo (as depicted in Figure S7).

### Regions of overlapping FCT indices findings

**Fig. 8:**
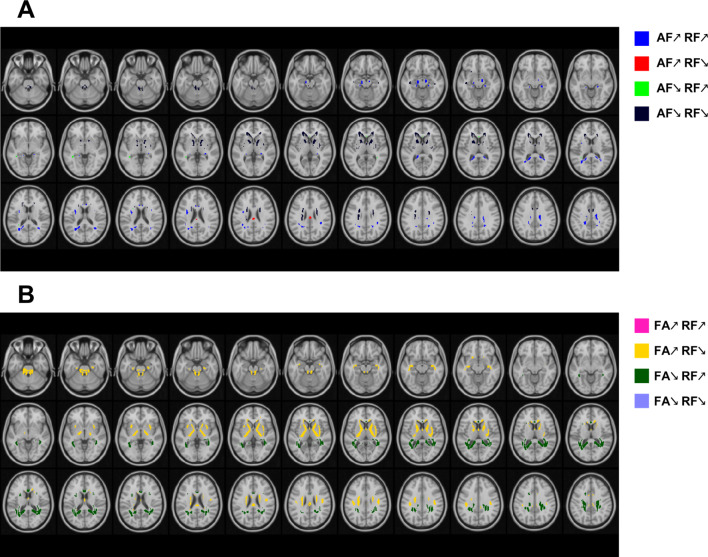
Axial representations of overlapping WM areas for positive and negative linear age effects of (**A**) axial FC (AF) and radial FC (RF) and (**B**) FA of FCT and RF. Same as previous voxel-wise maps, only the voxels that have passed significance test were plotted

We further investigated the areas revealing overlapping trends of different FCT indices with increasing age (as shown in Fig. 8). Relatively small overlapping areas were discovered for age effects on axial FC, in which reductions in axial FC coincided with negative age effects on radial FC in ALIC, ACR, SCBP. The results demonstrated that the pattern of the distribution of age effects on radial FC corresponded with the map of age effects on FA_FCT, in which age effects on FA_FCT are contrary to those of radial FC in most areas. It could be shown that FA_FCT reductions were accompanied by concomitant positive age effects on radial FC in areas in PCR, SLF, PTR, SCC, while other areas in WM, including FX, PLIC, ALIC, PCT, SCBP, revealed FA_FCT increments with radial FC decrements.

### Age effects on FCT indices in different sex groups

We further analyzed the age effects on FCT indices across different sex groups. On the one hand, we note that overall, female subjects exhibited significant age effects on more WM bundles compared to males. For example, regarding the age effects on FA of FCT, twenty-one WM tracts in the female group exhibited significant age effects, including bilateral ML, SCBP, ALIC, PCR, PTR, SLF, and TAP, among others (10 negative effects and 11 positive effects). In contrast, significant age effects were observed in 15 tracts for the male group. However, on the other hand, relevant analyses on the interaction effects between age and sex (as shown in the Table S3) indicated that there were no significant interactions between age and sex observed regarding each FCT index in all WM bundles. Meanwhile, there existed a consistency in the age-related effects on FCT indices between males and females. In WM bundles exhibiting significant age effects for two groups, both male and female groups revealed changes in the same direction (with no inconsistent signs), as shown in the Table S2.

## Discussion

This study investigated the effects of normal aging on the temporal correlations in resting state BOLD signals measured from brain WM. BOLD signals were acquired using fMRI from a selected group of healthy volunteers spanning a large age range, and FCTs were constructed voxel-wise for each subject, from which tensor indices were derived. Our voxel level analysis shows that there are both positive and negative age-related effects in tensor indices including FA_FCT, axial and radial FC. This dimorphic pattern, i.e., opposite directions of changes in the brain, also existed for the averaged FCT indices at the tract level. Our concomitant analysis of the tensor indices revealed that negative age effects on FA_FCT mostly corresponded to positive age effects on radial FC and vice versa.

As mentioned earlier, brain structural changes with age have been characterized by numerous previous studies. Most notably, reductions in FA of diffusion tensors have been widely observed and confirmed (Càmara et al., 2007; Hugenschmidt et al., 2008; Minati et al., 2007; Pfefferbaum et al., 2005; Zhang et al., 2010), reflecting age-related changes to brain tissue which decreases the anisotropy of water diffusion therein. In addition, distinct patterns of variations of other indices, including RD and AD, were also seen, each of which corresponds to certain biological processes (Burzynska et al., 2010; Song et al., 2002, 2003, 2005; Sun et al., 2006).

FCT and the corresponding index analysis provides a way to study age effects on the correlational anisotropy of BOLD variations in WM. Indices of FCT depict a detailed picture of correlational anisotropy in local functional signals. The FCT is estimated on the basis of correlations between a voxel and its neighbors in different directions, so eigenvalues of the FCT represent the degree of local functional correlations of the voxel along the corresponding characteristic directions. Axial FC, the largest eigenvalue of the FCT, then defines the correlation along the primary characteristic direction, and radial FC, the average value of the other two eigenvalues, then describes the correlation along the direction perpendicular to the primary one. Mean FC defines the average intensity of functional correlation corresponding to each feature direction. Additionally, FA of FCT gauges the overall directionality of local functional correlations.

It can be observed from our study that most areas in WM exhibited negative age-related effects on axial FC, decreasing functional correlations along the fiber tracts. Meanwhile, radial FC and mean FC also showed a decreasing trend in several areas in WM. The decrease in FCT indices in WM may be attributable to multiple factors. Firstly, it has been well recognized that BOLD signal changes are driven by the combined effects from cerebral blood flow (CBF), cerebral blood volume (CBV), and blood oxygenation (Buxton et al., 2004; Davis et al., 1998; Hoge et al., 1999). Previous works have demonstrated that there is a gradual decline in CBF (Mokhber et al., 2021), and age-related reductions in CBF occur throughout the brain parenchyma (Buijs et al., 1998; Chen et al., 2011; Claus et al., 1998; Kashimada et al., 1994; Leenders et al., 1990; Martin et al., 1991; Parkes et al., 2004; Shin et al., 2007; Stoquart-ElSankari et al., 2007). It has also been shown that there are positive correlations between regional CBF and FC in brain networks (Jann et al., 2015; Liang et al., 2013). Therefore, reduction in CBF may lead to changes of BOLD effects and FC measures during normal aging, and subsequently cause the decrease of FCT indices in WM. Secondly, WM microstructural changes in elderly populations may play important roles as well in the observed age-related variations in FCT indices. A body of DTI studies have demonstrated WM structural changes due to aging, such as fiber loss or demyelination, which may presumably be another driving factor for the observed decreases in FCT indices.

Besides the reduction of axial, radial and mean FC in WM during normal aging, positive age effects were also seen in several regions in WM, including PCR, SLF, PTR, etc., which revealed an evidently dimorphous pattern, rather than a whole-brain distributed structural degradation. The distribution of age effects on mean FC is comparable to the distribution of radial FC, indicating that radial FC might assume a dominant role in modulating the age effects on mean FC. First, potential functional compensation may be the cause of the positive age effects of axial and radial FC. It was reported earlier that positive age-related changes occurred in cortical and subcortical areas for both long- and short-range functional connectivity density (Tomasi & Volkow, 2012). In fact, preservations of brain networks have been found in normal aging (and in an AD group) (Reuter-Lorenz & Park, 2010; Stern et al., 2000), which may be attributable to compensations of processing deficits from the age effects. Additionally, positive age effects in parallel to reductions of FC indices are in keeping with non-uniform variations of cognitive performances during normal aging. For example, the cognitive functions that include attention, memory and executive function are affected the most by aging (Craik & Salthouse, 2011), while other functions, such as language and decision making, are relatively preserved (Sanfey & Hastie, 2000; Wingfield & Grossman, 2006).

Regarding the age effect on FA of FCT in reflecting the degree of functional anisotropy, its alterations might be related to two key microstructural factors, including myelination, or tract coherence. FA_FCT exhibited negative age effects in WM bundles including PCR, PTR, SLF, and SCC, etc. Previous studies have also indicated that the DTI FA of these tracts reveal negative correlations with age (Bendlin et al., 2010; Ota et al., 2006). Nevertheless, there are notable relative differences in the magnitudes of the age effects between the two measures. Among these, the age effect on DTI FA in SCC was the weakest among all parts of the corpus callosum (Bendlin et al., 2010), while the corresponding FCT FA exhibited the strongest correlation with age within SCC (the age effects on FCT FA in BCC and GCC were not significant), as shown in Figure S5. It is worth noting that a DTI study involving 200 subjects indicated that the DTI FA values of SCBP showed positive age effects and suggested the tract coherence plays an important role in adult lifespan (Kanaan et al., 2016), which provides evidence and insights into the positive age effects observed in the FCT FA of SCBP. Additionally, concerning other WM tracts (including ALIC, PLIC, and FX, etc.), their respective FCT FA values showed positive age-related correlations, while the corresponding DTI FA values still exhibit negative age effects (Jang et al., 2011; Kawaguchi et al., 2010). We should note that, while the FC indices corresponding to these WM tracts all displayed negative age correlations, the findings that the magnitudes of the negative age effects on radial FC were greater than those on the axial FC has resulted in the FCT FA still showing positive age effects. Furthermore, as mentioned previously, the WM tracts that show positive age effects in ReHo exhibit a similar pattern when compared to the corresponding bundles in FA of FCT. The observation of an increase in ReHo with increasing age has been noted previously and sparked meaningful discussions regarding compensatory mechanism (Montalà-Flaquer et al., 2023). Montalà-Flaquer et al. discovered that significantly higher ReHo values in the oldest age group ($\:\ge\:80$ years), suggesting a compensatory mechanism to offset functional deficits (Montalà-Flaquer et al., 2023). Our findings in this work provide further possible evidence for a potential compensatory effect, suggesting that specific brain regions might respond adaptively to aging.

As for the sex differences in the age effects on FCT properties, as mentioned earlier, we found that the female group showed significant age effects in more WM bundles than male subjects, while no age-by-sex interaction effects in FCT indices were observed, which might suggest the absence of sex difference in the aging process of anisotropic functional correlation in WM. Previous works have demonstrated no age-by-sex interaction effects in WM volume (Liu et al., 2016) and WM structural integrity (Hsu et al., 2010; Inano et al., 2011), while other studies also demonstrated significant interactions between sex and age in DTI indices (Abe et al., 2010; Kochunov et al., 2012). The results of our study, along with previous findings, further revealed the complexity of sex differences in aging process.

This research has studied age-related changes in FCT, which demonstrated both age-related reductions and increments of tensor indices of FCT at voxel- and bundle-levels. We note that, however, this work has a few limitations that need prudent considerations. Although the age effects on metrics of diffusion tensors for the corresponding data group and the related comparisons with FCT are not within the scope of this work, FCT offers a different perspective that helps enhance our understanding of the functional architecture of WM and its associated aging-related traits, which cannot be solved with diffusion imaging. Additionally, indeed, partial volume effect is an imperative factor to consider in MRI imaging. In this study, to mitigate the corresponding impact, all the analyses pertaining to the FCT indices in our study were specifically focused on the deep WM region. The JHU ICBM-DTI WM atlas we employed adopted a previously established parcellation map for the deep WM parcellation, which does not encompass the more superficially located WM that fills the space between the deep WM and the cortex (Mori et al., 2008; Oishi et al., 2008, 2009). Furthermore, previous studies have shown that the directional preferences observed in the functional correlation tensor in resting state demonstrate a degree of consistency with those revealed by diffusion tensors (Ding et al., 2016), but we need to recognize that the FCT relies on temporal correlations in BOLD signals between neighboring voxels. It has been widely acknowledged that BOLD signal fluctuations are intimately related to vascular phenomena, such as CBF and CBV (Buxton et al., 2004; Davis et al., 1998; Hoge et al., 1999). Therefore, indices of FCT might not be direct nor sole measures of neural activities, but rather combined effects from both vascular and neural events. Disentangling these components and subsequently conducting more precise analysis of age-related variations are thus an important question for future studies. Additionally, it is noted that the squares of correlation coefficients between fMRI signals of neighboring voxels were utilized as the degree of temporal correlations. While this is a feasible approach (Ding et al., 2013, 2016), the exploration of other measures for FCT construction is beyond the scope of this work. Also, in this study, the voxel size of the original data is $\:4\times\:4\times\:4$ mm. Within this voxel size, the presence of crossing/kissing tracts in WM structure cannot be ignored, and the potential impact on anisotropic functional correlations will need to be carefully investigated in future research. Despite these challenges, the results highlight how WM functional connectivity varies with normal aging in a region-specific and age-specific manner. Additionally, the study uncovers sex differences in the effects of aging, although no significant interaction between age and sex was found. The study has made the first attempt to employ FCT for quantitative characterizations of age-effects of spatiotemporal correlations in WM BOLD signals, which may provide novel insights into how the functional characteristics of WM evolve with brain aging.

## Electronic supplementary material

Below is the link to the electronic supplementary material.

Supplementary Material 1

## Data Availability

The dataset analyzed in this study is publicly available in the Open Access Series of Imaging Studies – stage 3 database (OASIS-3, https://www.oasis-brains.org).

## References

[CR1] Abe, O., Yamasue, H., Yamada, H., Masutani, Y., Kabasawa, H., Sasaki, H., Takei, K., Suga, M., Kasai, K., & Aoki, S. (2010). Sex dimorphism in gray/white matter volume and diffusion tensor during normal aging. *NMR in Biomedicine*, *23*(5), 446–458. 20310078 10.1002/nbm.1479

[CR2] Barrick, T. R., Charlton, R. A., Clark, C. A., & Markus, H. S. (2010). White matter structural decline in normal ageing: A prospective longitudinal study using tract-based spatial statistics. *Neuroimage*, *51*(2), 565–577. 20178850 10.1016/j.neuroimage.2010.02.033

[CR3] Bendlin, B. B., Fitzgerald, M. E., Ries, M. L., Xu, G., Kastman, E. K., Thiel, B. W., Rowley, H. A., Lazar, M., Alexander, A. L., & Johnson, S. C. (2010). White matter in aging and cognition: A cross-sectional study of microstructure in adults aged eighteen to eighty-three. *Developmental Neuropsychology*, *35*(3), 257–277. 20446132 10.1080/87565641003696775 PMC2895988

[CR4] Benjamini, Y., & Hochberg, Y. (1995). Controlling the false discovery rate: A practical and powerful approach to multiple testing. *Journal of the Royal Statistical Society: Series B (Methodological)*, *57*(1), 289–300.

[CR5] Buijs, P. C., Krabbe-Hartkamp, M. J., Bakker, C., de Lange, E. E., Ramos, L., Breteler, M., & Mali, W. (1998). Effect of age on cerebral blood flow: Measurement with ungated two-dimensional phase-contrast MR Angiography in 250 adults. *Radiology*, *209*(3), 667–674. 9844657 10.1148/radiology.209.3.9844657

[CR6] Burzynska, A. Z., Preuschhof, C., Bäckman, L., Nyberg, L., Li, S. C., Lindenberger, U., & Heekeren, H. R. (2010). Age-related differences in white matter microstructure: Region-specific patterns of diffusivity. *Neuroimage*, *49*(3), 2104–2112. 19782758 10.1016/j.neuroimage.2009.09.041

[CR7] Buxton, R. B., Uludağ, K., Dubowitz, D. J., & Liu, T. T. (2004). Modeling the hemodynamic response to brain activation. *Neuroimage*, *23*, S220–S233. 15501093 10.1016/j.neuroimage.2004.07.013

[CR8] Càmara, E., Bodammer, N., Rodríguez-Fornells, A., & Tempelmann, C. (2007). Age-related water diffusion changes in human brain: A voxel-based approach. *Neuroimage*, *34*(4), 1588–1599. 17188516 10.1016/j.neuroimage.2006.09.045

[CR9] Chen, J. J., Rosas, H. D., & Salat, D. H. (2011). Age-associated reductions in cerebral blood flow are independent from regional atrophy. *Neuroimage*, *55*(2), 468–478. 21167947 10.1016/j.neuroimage.2010.12.032 PMC3435846

[CR10] Chen, X., Zhang, H., Zhang, L., Shen, C., Lee, S. W., & Shen, D. (2017). Extraction of dynamic functional connectivity from brain grey matter and white matter for MCI classification. *Human Brain Mapping*, *38*(10), 5019–5034. 28665045 10.1002/hbm.23711 PMC5593789

[CR11] Claus, J. J., Breteler, M., Hasan, D., Krenning, E., Bots, M., Grobbee, D., Van Swieten, J., Van Harskamp, F., & Hofman, A. (1998). Regional cerebral blood flow and cerebrovascular risk factors in the elderly population. *Neurobiology of Aging*, *19*(1), 57–64. 9562504 10.1016/s0197-4580(98)00004-9

[CR12] Coupé, P., Catheline, G., Lanuza, E., Manjón, J. V., & Initiative, A. s. D. N. (2017). Towards a unified analysis of brain maturation and aging across the entire lifespan: A MRI analysis. *Human Brain Mapping*, *38*(11), 5501–5518. 28737295 10.1002/hbm.23743 PMC6866824

[CR13] Cox, S. R., Ritchie, S. J., Tucker-Drob, E. M., Liewald, D. C., Hagenaars, S. P., Davies, G., Wardlaw, J. M., Gale, C. R., Bastin, M. E., & Deary, I. J. (2016). Ageing and brain white matter structure in 3,513 UK Biobank participants. *Nature Communications*, *7*(1), 13629. 27976682 10.1038/ncomms13629 PMC5172385

[CR14] Craik, F. I., & Salthouse, T. A. (2011). *The handbook of aging and cognition*. Psychology.

[CR15] Davis, T. L., Kwong, K. K., Weisskoff, R. M., & Rosen, B. R. (1998). Calibrated functional MRI: mapping the dynamics of oxidative metabolism. *Proceedings of the National Academy of Sciences*, *95* (4), 1834–1839. 10.1073/pnas.95.4.1834 PMC19199 9465103

[CR16] DeCarli, C., Massaro, J., Harvey, D., Hald, J., Tullberg, M., Au, R., Beiser, A., D’Agostino, R., & Wolf, P. A. (2005). Measures of brain morphology and infarction in the framingham heart study: Establishing what is normal. *Neurobiology of Aging*, *26*(4), 491–510. 10.1016/j.neurobiolaging.2004.05.004 15653178 10.1016/j.neurobiolaging.2004.05.004

[CR18] Ding, Z., Newton, A. T., Xu, R., Anderson, A. W., Morgan, V. L., & Gore, J. C. (2013). Spatio-temporal correlation tensors reveal functional structure in human brain. *PloS One*, 8 (12), e82107. 10.1371/journal.pone.0082107 PMC3855380 24339997

[CR19] Ding, Z., Xu, R., Bailey, S. K., Wu, T. L., Morgan, V. L., Cutting, L. E., Anderson, A. W., & Gore, J. C. (2016). Visualizing functional pathways in the human brain using correlation tensors and magnetic resonance imaging. *Magnetic Resonance Imaging*, *34*(1), 8–17. 26477562 10.1016/j.mri.2015.10.003 PMC4714593

[CR17] Ding, Z., Huang, Y., K Bailey, S., Gao, Y., E Cutting, L., P Rogers, B., T Newton, A., & C Gore, J. (2018). Detection of synchronous brain activity in white matter tracts at rest and under functional loading. *Proceedings of the National Academy of Sciences*, *115*(3), 595–600. 10.1073/pnas.1711567115 PMC5776967 29282320

[CR21] Gao, Y., Sengupta, A., Li, M., Zu, Z., Rogers, B. P., Anderson, A. W., Ding, Z., Gore, J. C., & Initiative, A. (2020). Functional connectivity of white matter as a biomarker of cognitive decline in Alzheimer’s disease. *PloS One*, *15*(10), e0240513. s. D. N. 33064765 10.1371/journal.pone.0240513 PMC7567362

[CR20] Gao, Y., Li, M., Huang, A. S., Anderson, A. W., Ding, Z., Heckers, S. H., Woodward, N. D., & Gore, J. C. (2021). Lower functional connectivity of white matter during rest and working memory tasks is associated with cognitive impairments in schizophrenia. *Schizophrenia Research*, *233*, 101–110. 34215467 10.1016/j.schres.2021.06.013 PMC8442250

[CR22] Gao, Y., Zhao, Y., Li, M., Lawless, R. D., Schilling, K. G., Xu, L., Shafer, A. T., Beason-Held, L. L., Resnick, S. M., & Rogers, B. P. (2023). Functional alterations in bipartite network of white and grey matters during aging. *Neuroimage*, *278*, 120277. 37473978 10.1016/j.neuroimage.2023.120277 PMC10529380

[CR23] Gaser, C., Dahnke, R., Thompson, P. M., Kurth, F., & Luders, E. (2022). CAT-a computational anatomy toolbox for the analysis of structural MRI data. *BioRxiv*, 2022.2006. 2011.495736. 10.1093/gigascience/giae049 PMC11299546 39102518

[CR24] Gawryluk, J. R., Mazerolle, E. L., & D’Arcy, R. C. (2014). Does functional MRI detect activation in white matter? A review of emerging evidence, issues, and future directions. *Frontiers in Neuroscience*, *8*, 101955. 10.3389/fnins.2014.00239 PMC4125856 25152709

[CR25] Gore, J. C., Li, M., Gao, Y., Wu, T. L., Schilling, K. G., Huang, Y., Mishra, A., Newton, A. T., Rogers, B. P., & Chen, L. M. (2019). Functional MRI and resting state connectivity in white matter-a mini-review. *Magnetic Resonance Imaging*, *63*, 1–11. 31376477 10.1016/j.mri.2019.07.017 PMC6861686

[CR26] Helenius, J., Perkiö, J., Soinne, L., Østergaard, L., Carano, R. A., Salonen, O., Savolainen, S., Kaste, M., Aronen, H. J., & Tatlisumak, T. (2003). Cerebral hemodynamics in a healthy population measured by dynamic susceptibility contrast MR imaging. *Acta Radiologica*, *44*(5), 538–546. 14510762 10.1080/j.1600-0455.2003.00104.x

[CR27] Hoge, R. D., Atkinson, J., Gill, B., Crelier, G. R., Marrett, S., & Pike, G. B. (1999). Linear coupling between cerebral blood flow and oxygen consumption in activated human cortex. *Proceedings of the National Academy of Sciences*, *96* (16), 9403–9408. 10.1073/pnas.96.16.9403 PMC17795 10430955

[CR28] Hsu, J. L., Van Hecke, W., Bai, C. H., Lee, C. H., Tsai, Y. F., Chiu, H. C., Jaw, F. S., Hsu, C. Y., Leu, J. G., & Chen, W. H. (2010). Microstructural white matter changes in normal aging: A diffusion tensor imaging study with higher-order polynomial regression models. *Neuroimage*, *49*(1), 32–43. 19699804 10.1016/j.neuroimage.2009.08.031

[CR29] Huang, Y., Bailey, S. K., Wang, P., Cutting, L. E., Gore, J. C., & Ding, Z. (2018). Voxel-wise detection of functional networks in white matter. *Neuroimage*, *183*, 544–552. 30144573 10.1016/j.neuroimage.2018.08.049 PMC6226032

[CR30] Hugenschmidt, C. E., Peiffer, A. M., Kraft, R. A., Casanova, R., Deibler, A. R., Burdette, J. H., Maldjian, J. A., & Laurienti, P. J. (2008). Relating imaging indices of white matter integrity and volume in healthy older adults. *Cerebral Cortex*, *18*(2), 433–442. 17575289 10.1093/cercor/bhm080

[CR31] Inano, S., Takao, H., Hayashi, N., Abe, O., & Ohtomo, K. (2011). Effects of age and gender on white matter integrity. *American Journal of Neuroradiology*, *32*(11), 2103–2109. 21998104 10.3174/ajnr.A2785 PMC7964377

[CR32] Jang, S. H., Cho, S. H., & Chang, M. C. (2011). Age-related degeneration of the fornix in the human brain: A diffusion tensor imaging study. *International Journal of Neuroscience*, *121*(2), 94–100. 21062216 10.3109/00207454.2010.531894

[CR33] Jann, K., Gee, D. G., Kilroy, E., Schwab, S., Smith, R. X., Cannon, T. D., & Wang, D. J. (2015). Functional connectivity in BOLD and CBF data: Similarity and reliability of resting brain networks. *Neuroimage*, *106*, 111–122. 25463468 10.1016/j.neuroimage.2014.11.028 PMC4285775

[CR34] Jockwitz, C., & Caspers, S. (2021). Resting-state networks in the course of aging—differential insights from studies across the lifespan vs. amongst the old. *Pflügers Archiv-European Journal of Physiology*, *473*, 793–803. 33576851 10.1007/s00424-021-02520-7 PMC8076139

[CR35] Kanaan, R. A., Allin, M., Picchioni, M. M., Shergill, S. S., & McGuire, P. K. (2016). White matter microstructural organization is higher with age in adult superior cerebellar peduncles. *Frontiers in Aging Neuroscience*, *8*, 71. 27148043 10.3389/fnagi.2016.00071 PMC4830843

[CR36] Kashimada, A., Machida, K., Honda, N., Mamiya, T., Takahashi, T., Kamano, T., Inoue, Y., & Osada, H. (1994). Measurement of cerebral blood flow in normal subjects by phase contrast MR imaging. *Nihon Igaku Hoshasen Gakkai Zasshi Nippon Acta Radiologica*, *54*(12), 1116–1125. 9261191

[CR37] Kawaguchi, H., Obata, T., Ota, M., Akine, Y., Ito, H., Ikehira, H., Kanno, I., & Suhara, T. (2010). Regional heterogeneity and age-related change in sub-regions of internal capsule evaluated by diffusion tensor imaging. *Brain Research*, *1354*, 30–39. 20682306 10.1016/j.brainres.2010.07.084

[CR38] Kochunov, P., Williamson, D., Lancaster, J., Fox, P., Cornell, J., Blangero, J., & Glahn, D. (2012). Fractional anisotropy of water diffusion in cerebral white matter across the lifespan. *Neurobiology of Aging*, *33*(1), 9–20. 20122755 10.1016/j.neurobiolaging.2010.01.014 PMC2906767

[CR39] Koen, J. D., & Rugg, M. D. (2019). Neural dedifferentiation in the aging brain. *Trends in Cognitive Sciences*, *23*(7), 547–559. 31174975 10.1016/j.tics.2019.04.012 PMC6635135

[CR40] Kong, Y., Niu, S., Gao, H., Yue, Y., Shu, H., Xie, C., Zhang, Z., & Yuan, Y. (2022). Multi-stage graph fusion networks for major depressive disorder diagnosis. *IEEE Transactions on Affective Computing*, *13*(4), 1917–1928.

[CR41] LaMontagne, P. J., Benzinger, T. L., Morris, J. C., Keefe, S., Hornbeck, R., Xiong, C., Grant, E., Hassenstab, J., Moulder, K., & Vlassenko, A. G. (2019). OASIS-3: longitudinal neuroimaging, clinical, and cognitive dataset for normal aging and Alzheimer disease. *MedRxiv*, 2019.2012. 2013.19014902.

[CR42] Lee, J., & Kim, H. J. (2022). Normal aging induces changes in the brain and neurodegeneration progress: Review of the structural, biochemical, metabolic, cellular, and molecular changes. *Frontiers in Aging Neuroscience*, *14*, 931536. 35847660 10.3389/fnagi.2022.931536 PMC9281621

[CR43] Leenders, K., Perani, D., Lammertsma, A., Heather, J., Buckingham, P., Jones, T., Healy, M., Gibbs, J., Wise, R., & Hatazawa, J. (1990). Cerebral blood flow, blood volume and oxygen utilization: Normal values and effect of age. *Brain*, *113*(1), 27–47. 2302536 10.1093/brain/113.1.27

[CR45] Li, M., Newton, A. T., Anderson, A. W., Ding, Z., & Gore, J. C. (2019). Characterization of the hemodynamic response function in white matter tracts for event-related fMRI. *Nature communications*, *10* (1), 1140. 10.1038/s41467-019-09076-2 PMC6408456 30850610

[CR44] Li, M., Gao, Y., Lawless, R. D., Xu, L., Zhao, Y., Schilling, K. G., Ding, Z., Anderson, A. W., Landman, B. A., & Gore, J. C. (2023). Changes in white matter functional networks across late adulthood. *Frontiers in aging neuroscience*, *15*. 10.3389/fnagi.2023.1204301 PMC10347529 37455933

[CR46] Liang, X., Zou, Q., He, Y., & Yang, Y. (2013). Coupling of functional connectivity and regional cerebral blood flow reveals a physiological basis for network hubs of the human brain. *Proceedings of the National Academy of Sciences*, *110*(5), 1929–1934. 10.1073/pnas.1214900110 PMC3562840 23319644

[CR47] Liu, H., Wang, L., Geng, Z., Zhu, Q., Song, Z., Chang, R., & Lv, H. (2016). A voxel-based morphometric study of age-and sex-related changes in white matter volume in the normal aging brain. *Neuropsychiatric Disease and Treatment*, 453–465. 10.2147/NDT.S90674 PMC4771405 26966366

[CR48] Lodish, H., Berk, A., Zipursky, S. L., Matsudaira, P., Baltimore, D., & Darnell, J. (2000). Neurotransmitters, synapses, and impulse transmission. In *Molecular Cell Biology. 4th edition*. WH Freeman.

[CR49] Lu, T., Pan, Y., Kao, S. Y., Li, C., Kohane, I., Chan, J., & Yankner, B. A. (2004). Gene regulation and DNA damage in the ageing human brain. *Nature*, *429*(6994), 883–891. 15190254 10.1038/nature02661

[CR50] Martin, A. J., Friston, K. J., Colebatch, J. G., & Frackowiak, R. S. (1991). Decreases in regional cerebral blood flow with normal aging. *Journal of Cerebral Blood Flow & Metabolism*, *11*(4), 684–689. 2050757 10.1038/jcbfm.1991.121

[CR51] Mattson, M. P., & Arumugam, T. V. (2018). Hallmarks of brain aging: Adaptive and pathological modification by metabolic states. *Cell Metabolism*, *27*(6), 1176–1199. 29874566 10.1016/j.cmet.2018.05.011 PMC6039826

[CR52] Minati, L., Grisoli, M., & Bruzzone, M. (2007). MR spectroscopy, functional MRI, and diffusion-tensor imaging in the aging brain: A conceptual review. *Journal of Geriatric Psychiatry and Neurology*, *20*(1), 3–21. 17341766 10.1177/0891988706297089

[CR53] Mokhber, N., Shariatzadeh, A., Avan, A., Saber, H., Babaei, G. S., Chaimowitz, G., & Azarpazhooh, M. R. (2021). Cerebral blood flow changes during aging process and in cognitive disorders: A review. *The Neuroradiology Journal*, *34*(4), 300–307. 33749402 10.1177/19714009211002778 PMC8447819

[CR54] Montalà-Flaquer, M., Cañete-Massé, C., Vaqué-Alcázar, L., Bartrés-Faz, D., Peró-Cebollero, M., & Guàrdia-Olmos, J. (2023). Spontaneous brain activity in healthy aging: An overview through fluctuations and regional homogeneity. *Frontiers in Aging Neuroscience*, *14*, 1002811. 36711210 10.3389/fnagi.2022.1002811 PMC9877451

[CR55] Mori, S., Oishi, K., Jiang, H., Jiang, L., Li, X., Akhter, K., Hua, K., Faria, A. V., Mahmood, A., & Woods, R. (2008). Stereotaxic white matter atlas based on diffusion tensor imaging in an ICBM template. *Neuroimage*, *40*(2), 570–582. 18255316 10.1016/j.neuroimage.2007.12.035 PMC2478641

[CR56] Morrison, J. H., & Hof, P. R. (1997). Life and death of neurons in the aging brain. *Science*, *278*(5337), 412–419. 9334292 10.1126/science.278.5337.412

[CR57] Nikhra, V. (2017). The aging brain: Recent research and concepts. *Gerontol Geriatr Stud*, *1*, 1–11.

[CR59] Oishi, K., Zilles, K., Amunts, K., Faria, A., Jiang, H., Li, X., Akhter, K., Hua, K., Woods, R., & Toga, A. W. (2008). Human brain white matter atlas: Identification and assignment of common anatomical structures in superficial white matter. *Neuroimage*, *43*(3), 447–457. 18692144 10.1016/j.neuroimage.2008.07.009 PMC2586008

[CR58] Oishi, K., Faria, A., Jiang, H., Li, X., Akhter, K., Zhang, J., Hsu, J. T., Miller, M. I., van Zijl, P. C., & Albert, M. (2009). Atlas-based whole brain white matter analysis using large deformation diffeomorphic metric mapping: Application to normal elderly and Alzheimer’s disease participants. *Neuroimage*, *46*(2), 486–499. 19385016 10.1016/j.neuroimage.2009.01.002 PMC2885858

[CR60] Ota, M., Obata, T., Akine, Y., Ito, H., Ikehira, H., Asada, T., & Suhara, T. (2006). Age-related degeneration of corpus callosum measured with diffusion tensor imaging. *Neuroimage*, *31*(4), 1445–1452. 16563802 10.1016/j.neuroimage.2006.02.008

[CR61] Park, D. C., & Reuter-Lorenz, P. (2009). The adaptive brain: Aging and neurocognitive scaffolding. *Annual Review of Psychology*, *60*, 173–196. 19035823 10.1146/annurev.psych.59.103006.093656 PMC3359129

[CR62] Parkes, L. M., Rashid, W., Chard, D. T., & Tofts, P. S. (2004). Normal cerebral perfusion measurements using arterial spin labeling: Reproducibility, stability, and age and gender effects. *Magnetic Resonance in Medicine: An Official Journal of the International Society for Magnetic Resonance in Medicine*, *51*(4), 736–743. 10.1002/mrm.20023 15065246

[CR63] Penny, W. D., Friston, K. J., Ashburner, J. T., Kiebel, S. J., & Nichols, T. E. (2011). *Statistical parametric mapping: The analysis of functional brain images*. Elsevier.

[CR64] Peters, R. (2006). Ageing and the brain: This article is part of a series on ageing edited by Professor Chris Bulpitt. *Postgraduate Medical Journal*, *82*(964), 84–88. 10.1136/pgmj.2005.036665 16461469 10.1136/pgmj.2005.036665 PMC2596698

[CR65] Pfefferbaum, A., Adalsteinsson, E., & Sullivan, E. V. (2005). Frontal circuitry degradation marks healthy adult aging: Evidence from diffusion tensor imaging. *Neuroimage*, *26*(3), 891–899. 15955499 10.1016/j.neuroimage.2005.02.034

[CR66] Pierpaoli, C., & Basser, P. J. (1996). Toward a quantitative assessment of diffusion anisotropy. *Magnetic Resonance in Medicine*, *36*(6), 893–906. 8946355 10.1002/mrm.1910360612

[CR67] Pierpaoli, C., Jezzard, P., Basser, P. J., Barnett, A., & Di Chiro, G. (1996). Diffusion tensor MR imaging of the human brain. *Radiology*, *201*(3), 637–648. 8939209 10.1148/radiology.201.3.8939209

[CR68] Power, J. D., Barnes, K. A., Snyder, A. Z., Schlaggar, B. L., & Petersen, S. E. (2012). Spurious but systematic correlations in functional connectivity MRI networks arise from subject motion. *Neuroimage*, *59*(3), 2142–2154. 22019881 10.1016/j.neuroimage.2011.10.018 PMC3254728

[CR69] Raichle, M. E., MacLeod, A. M., Snyder, A. Z., Powers, W. J., Gusnard, D. A., & Shulman, G. L. (2001). A default mode of brain function. *Proceedings of the National Academy of Sciences*, *98* (2), 676–682. 10.1073/pnas.98.2.676 PMC14647 11209064

[CR70] Reuter-Lorenz, P. A. (2002). New visions of the aging mind and brain. *Trends in Cognitive Sciences*, *6*(9), 394–400. 12200182 10.1016/s1364-6613(02)01957-5

[CR71] Reuter-Lorenz, P. A., & Park, D. C. (2010). Human neuroscience and the aging mind: A new look at old problems. *Journals of Gerontology Series B: Psychological Sciences and Social Sciences*, *65*(4), 405–415. 20478901 10.1093/geronb/gbq035 PMC2883872

[CR72] Reuter-Lorenz, P. A., & Park, D. C. (2014). How does it STAC up? Revisiting the scaffolding theory of aging and cognition. *Neuropsychology Review*, *24*, 355–370. 25143069 10.1007/s11065-014-9270-9 PMC4150993

[CR73] Rostrup, E., Law, I., Blinkenberg, M., Larsson, H., Born, A. P., Holm, S., & Paulson, O. (2000). Regional differences in the CBF and BOLD responses to hypercapnia: A combined PET and fMRI study. *Neuroimage*, *11*(2), 87–97. 10679182 10.1006/nimg.1999.0526

[CR74] Sanfey, A. G., & Hastie, R. (2000). *Judgment and decision making across the adult life span*. A tutorial review of psychological research.

[CR75] Shin, W., Horowitz, S., Ragin, A., Chen, Y., Walker, M., & Carroll, T. J. (2007). Quantitative cerebral perfusion using dynamic susceptibility contrast MRI: Evaluation of reproducibility and age-and gender‐dependence with fully automatic image postprocessing algorithm. *Magnetic Resonance in Medicine: An Official Journal of the International Society for Magnetic Resonance in Medicine*, *58*(6), 1232–1241. 10.1002/mrm.21420 17969025

[CR77] Song, S. K., Sun, S. W., Ramsbottom, M. J., Chang, C., Russell, J., & Cross, A. H. (2002). Dysmyelination revealed through MRI as increased radial (but unchanged axial) diffusion of water. *Neuroimage*, *17*(3), 1429–1436. 12414282 10.1006/nimg.2002.1267

[CR76] Song, S. K., Sun, S. W., Ju, W. K., Lin, S. J., Cross, A. H., & Neufeld, A. H. (2003). Diffusion tensor imaging detects and differentiates axon and myelin degeneration in mouse optic nerve after retinal ischemia. *Neuroimage*, *20*(3), 1714–1722. 14642481 10.1016/j.neuroimage.2003.07.005

[CR78] Song, S. K., Yoshino, J., Le, T. Q., Lin, S. J., Sun, S. W., Cross, A. H., & Armstrong, R. C. (2005). Demyelination increases radial diffusivity in corpus callosum of mouse brain. *Neuroimage*, *26*(1), 132–140. 15862213 10.1016/j.neuroimage.2005.01.028

[CR79] Stern, Y., Moeller, J. R., Anderson, K. E., Luber, B., Zubin, N. R., DiMauro, A., Park, A., Campbell, C. E., Marder, K., & Bell, K. (2000). Different brain networks mediate task performance in normal aging and AD: Defining compensation. *Neurology*, *55*(9), 1291–1297. 11087770 10.1212/wnl.55.9.1291

[CR80] Stoquart-ElSankari, S., Balédent, O., Gondry-Jouet, C., Makki, M., Godefroy, O., & Meyer, M. E. (2007). Aging effects on cerebral blood and cerebrospinal fluid flows. *Journal of Cerebral Blood Flow & Metabolism*, *27*(9), 1563–1572. 17311079 10.1038/sj.jcbfm.9600462

[CR81] Sullivan, E. V., Rohlfing, T., & Pfefferbaum, A. (2010). Longitudinal study of callosal microstructure in the normal adult aging brain using quantitative DTI fiber tracking. *Developmental Neuropsychology*, *35*(3), 233–256. 20446131 10.1080/87565641003689556 PMC2867078

[CR82] Sun, S. W., Liang, H. F., Trinkaus, K., Cross, A. H., Armstrong, R. C., & Song, S. K. (2006). Noninvasive detection of cuprizone induced axonal damage and demyelination in the mouse corpus callosum. *Magnetic Resonance in Medicine: An Official Journal of the International Society for Magnetic Resonance in Medicine*, *55*(2), 302–308. 10.1002/mrm.20774 16408263

[CR83] Tomasi, D., & Volkow, N. D. (2012). Aging and functional brain networks. *Molecular Psychiatry*, *17*(5), 549–558. 10.1038/mp.2011.81 PMC3193908 21727896

[CR84] Walhovd, K. B., Westlye, L. T., Amlien, I., Espeseth, T., Reinvang, I., Raz, N., Agartz, I., Salat, D. H., Greve, D. N., & Fischl, B. (2011). Consistent neuroanatomical age-related volume differences across multiple samples. *Neurobiology of Aging*, *32*(5), 916–932. 19570593 10.1016/j.neurobiolaging.2009.05.013 PMC4040218

[CR85] Wang, J., Yang, Z., Zhang, M., Shan, Y., Rong, D., Ma, Q., Liu, H., Wu, X., Li, K., & Ding, Z. (2019). Disrupted functional connectivity and activity in the white matter of the sensorimotor system in patients with pontine strokes. *Journal of Magnetic Resonance Imaging*, *49*(2), 478–486. 30291655 10.1002/jmri.26214

[CR86] Wingfield, A., & Grossman, M. (2006). Language and the aging brain: Patterns of neural compensation revealed by functional brain imaging. *Journal of Neurophysiology*, *96*(6), 2830–2839. 17110737 10.1152/jn.00628.2006

[CR87] Xu, L., Choi, S., Zhao, Y., Li, M., Rogers, B. P., Anderson, A., Gore, J. C., Gao, Y., & Ding, Z. (2023). Seasonal variations of functional connectivity of human brains. *Scientific Reports*, *13*(1), 16898. 37803105 10.1038/s41598-023-43152-4 PMC10558480

[CR88] Yan, C. G., Wang, X. D., Zuo, X. N., & Zang, Y. F. (2016). DPABI: Data processing & analysis for (resting-state) brain imaging. *Neuroinformatics*, *14*, 339–351. 27075850 10.1007/s12021-016-9299-4

[CR89] Yankner, B. A., Lu, T., & Loerch, P. (2008). The aging brain. *Annu Rev Pathol Mech Dis*, *3*, 41–66. 10.1146/annurev.pathmechdis.2.010506.092044 18039130

[CR90] Zang, Y., Jiang, T., Lu, Y., He, Y., & Tian, L. (2004). Regional homogeneity approach to fMRI data analysis. *Neuroimage*, *22*(1), 394–400. 15110032 10.1016/j.neuroimage.2003.12.030

[CR91] Zhang, Y., Du, A. T., Hayasaka, S., Jahng, G., Hlavin, J., Zhan, W., Weiner, M. W., & Schuff, N. (2010). Patterns of age-related water diffusion changes in human brain by concordance and discordance analysis. *Neurobiology of Aging*, *31*(11), 1991–2001. 19036473 10.1016/j.neurobiolaging.2008.10.009 PMC2888604

