# Supplementary material for: Functional correlation tensors in brain white matter and the effects of normal aging
Source: Brain Imaging Behav. Author manuscript; Available in PMC 2025 Jan 7. (PMC11582213; doi:10.1007/s11682-024-00914-6)
Supplement: Supplementary Material [file NIHMS2033529-supplement-Supplementary_Material.pdf]

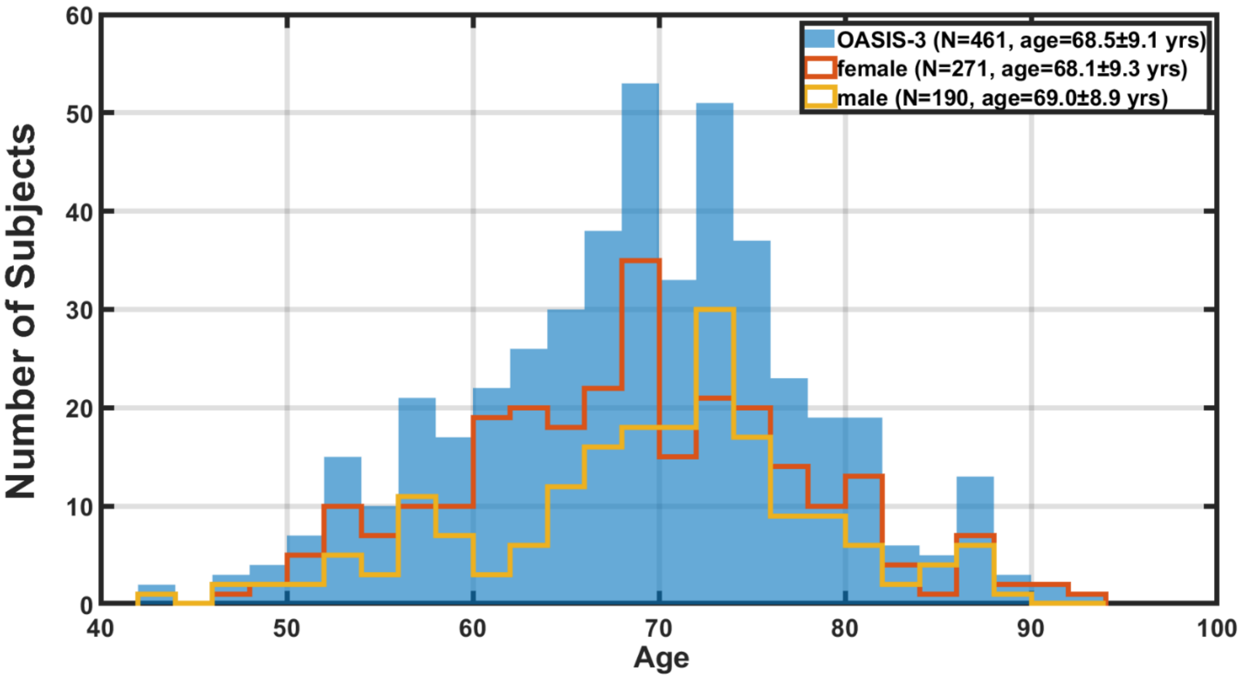

Figure S1. Histogram by age of OASIS-3 subjects (along with the histograms by age of female and male subjects) analyzed in this study.

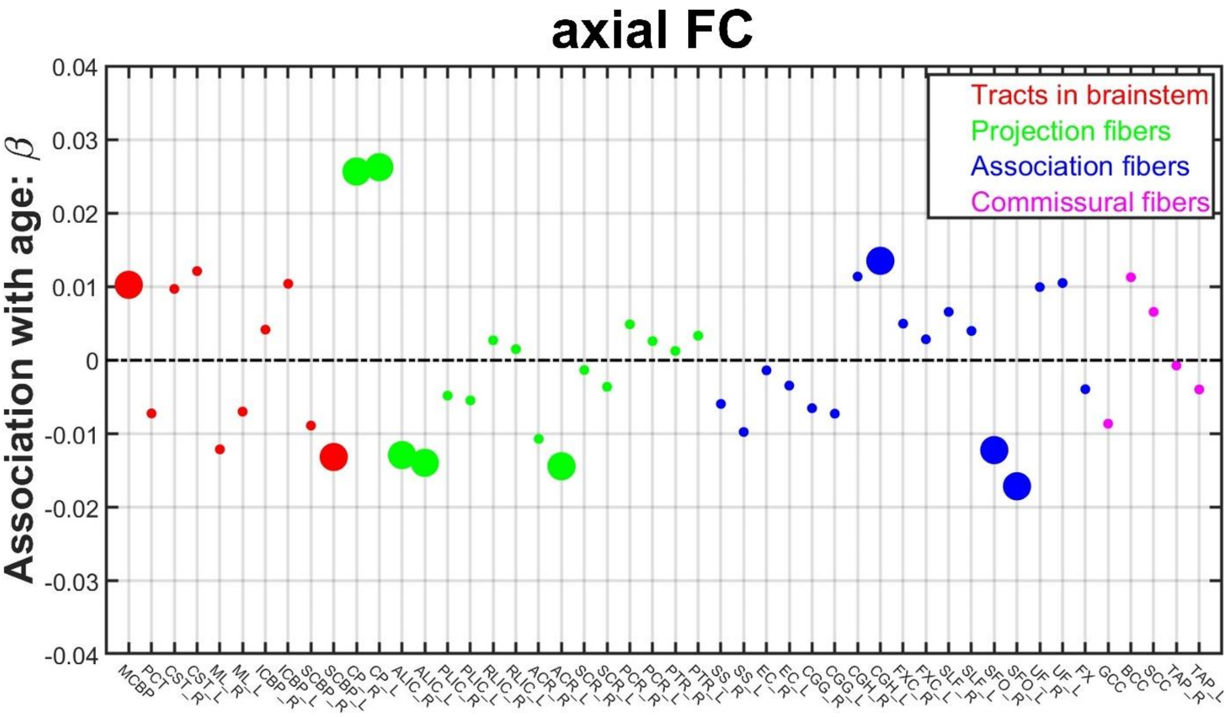

Figure S2. Age association β with axial FC of all WM tracts reported in this work. The red, green, blue, and magenta dots represent tracts in the brainstem, projection fibers, association fibers and commissural fibers respectively. The relatively bigger dots in this figure represent the WM tracts that revealed significant age associations with axial FC ($q < 0.05$).

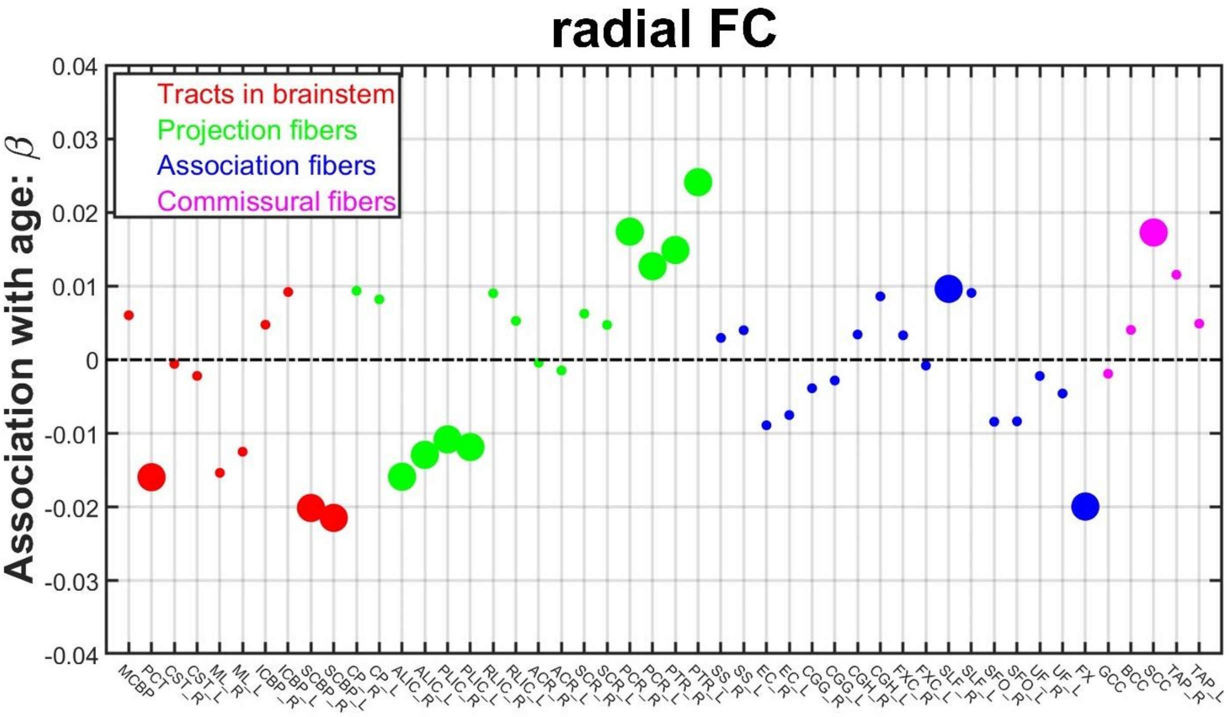

Figure S3. Age association β with radial FC of all WM tracts reported in this work. The red, green, blue, and magenta dots represent tracts in the brainstem, projection fibers, association fibers and commissural fibers respectively. The relatively bigger dots in this figure represent the WM tracts that revealed significant age associations with radial FC ($q < 0.05$).

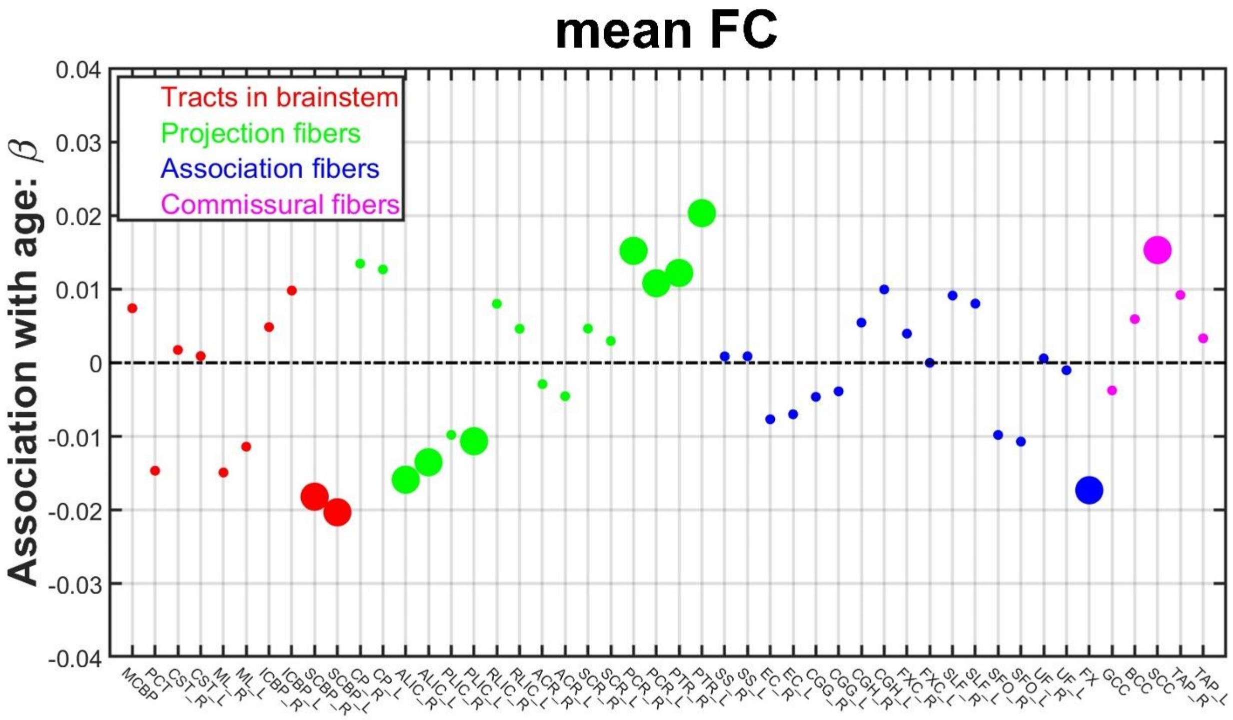

Figure S4. Age association β with mean FC of all WM tracts reported in this work. The red, green, blue, and magenta dots represent tracts in the brainstem, projection fibers, association fibers and commissural fibers respectively. The relatively bigger dots in this figure represent the WM tracts that revealed significant age associations with mean FC ($q < 0.05$).

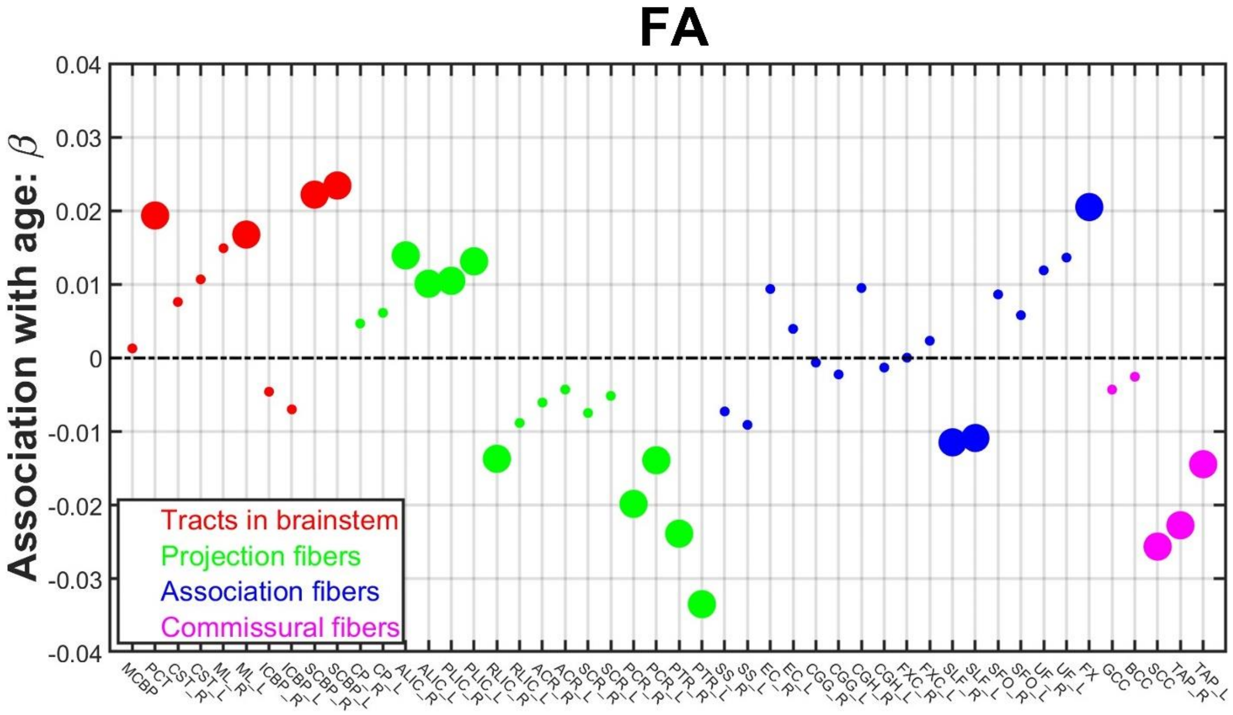

Figure S5. Age association β with FA of all WM tracts reported in this work. The red, green, blue, and magenta dots represent tracts in the brainstem, projection fibers, association fibers and commissural fibers respectively. The relatively bigger dots in this figure represent the WM tracts that revealed significant age associations with FA ($q < 0.05$).

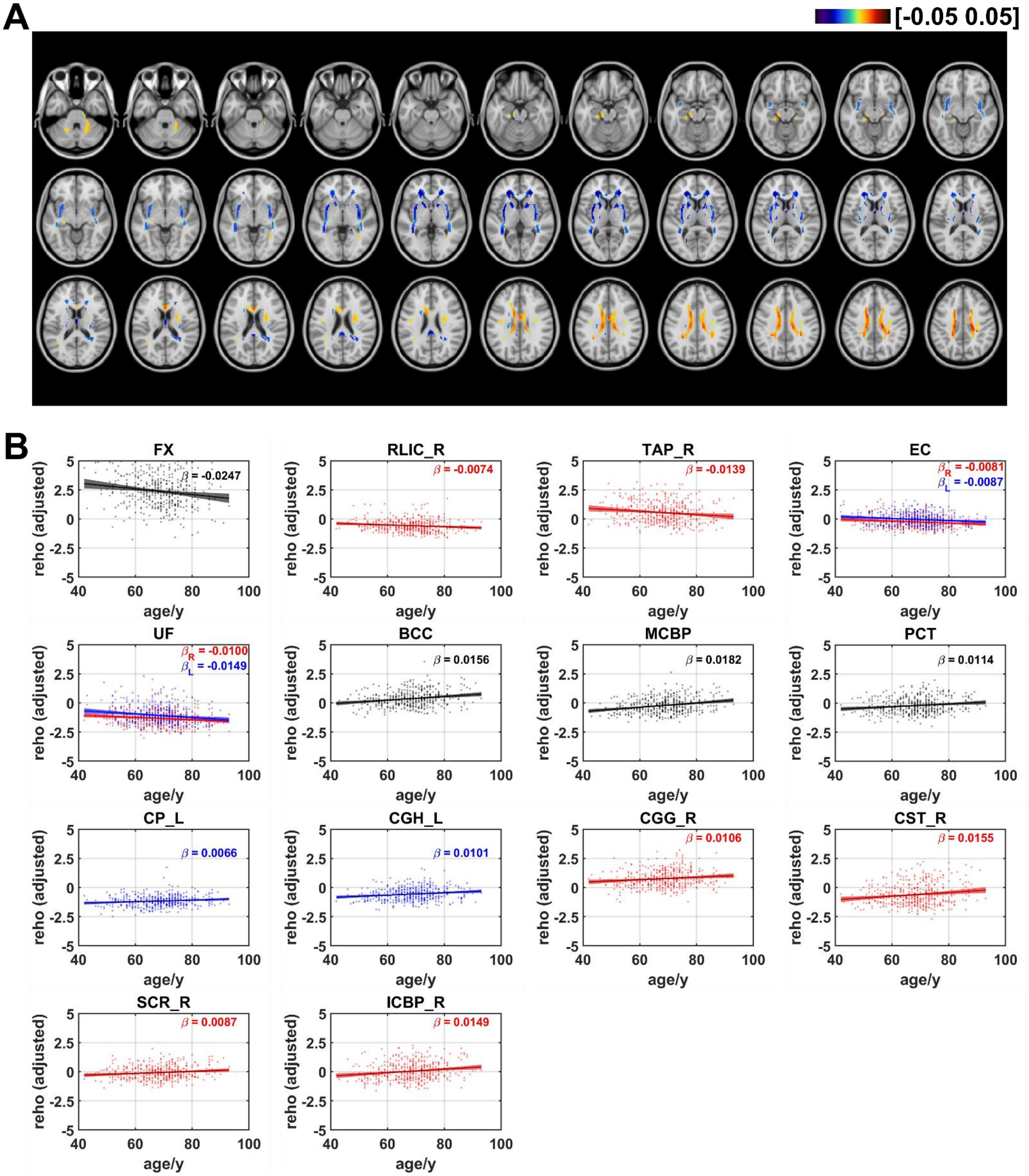

Figure S6. Age-related coefficients of ReHo. **(A)** Voxel-wise map of linear age effects on adjusted ReHo. All p values for the t-statistic test have been FDR corrected using Benjamini and Hochberg's method. Only the voxels that passed significance test were plotted. **(B)** Plots of significant age-related relationships between adjusted ReHo and age for different WM tracts. Age effects of ReHo of WM tracts in left and right hemisphere are plotted in blue and red respectively. Same as the voxel-wise analysis, all p-values for t-statistic test have been corrected and q-values were generated.

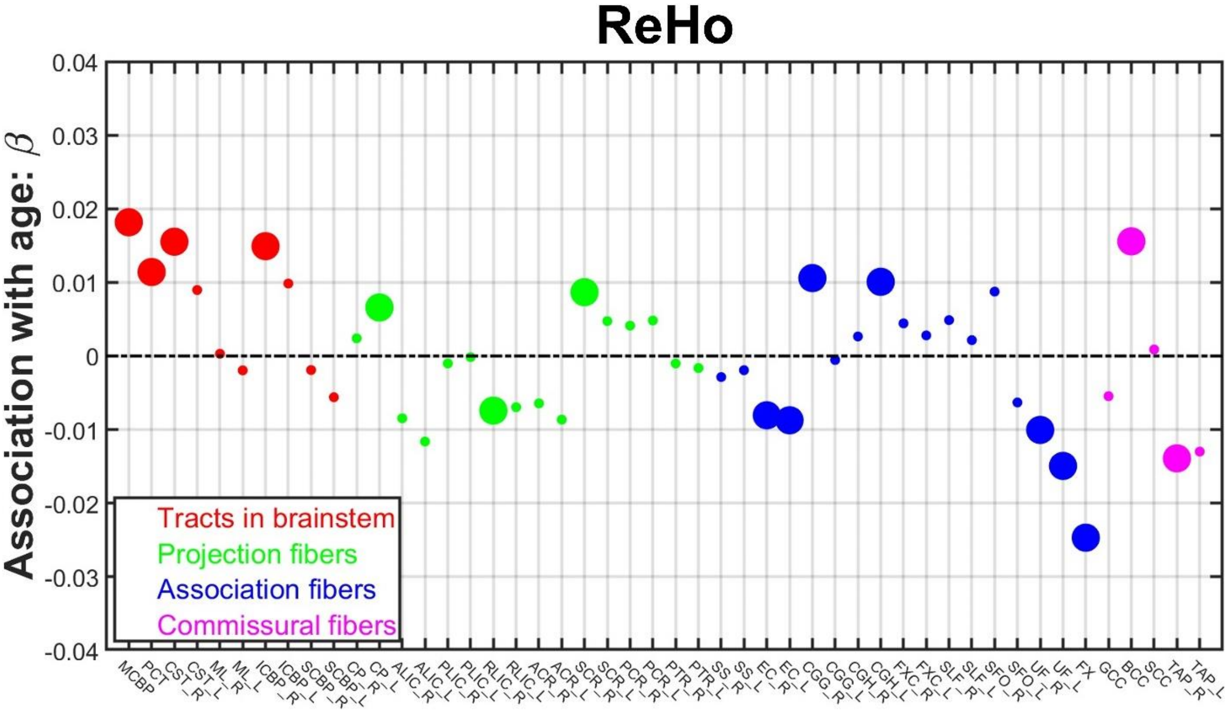

Figure S7. Age association β with ReHo of all WM tracts reported in this work. The red, green, blue, and magenta dots represent tracts in the brainstem, projection fibers, association fibers and commissural fibers respectively. The relatively bigger dots in this figure represent the WM tracts that revealed significant age associations with ReHo ($q < 0.05$).

Table S1. Summary of the number of subjects scanned in each site/scanner.

Site/Scanner Label	Number of Subjects	Vendor	fMRI			Age	
			Resolution (mm)	Matrix size	TR/TE (ms)	Range (y)	Average age (y)
OASIS3_dev35177	89	Siemens	4x4x4	64x64x36x164	2200/27	[57.0, 95.0]	73.9±7.5
OASIS3_dev35248	276	Siemens	4x4x4	64x64x36x164	2200/27	[42.0, 93.0]	65.7±8.9
OASIS3_dev51010	96	Siemens	4x4x4	64x64x36x164	2200/27	[46.0, 90.0]	71.3±8.2

Table S2. Coefficients β and q values of linear age-effects of axial FC, radial FC, mean FC, and FA_FCT averaged for WM tracts for both male and female groups, in which significant results are indicated in bold (significant level = 0.05, uncorrected). The subscripts 'male' and 'female' for β represent the age effect coefficients for the male and female groups, respectively.

WM tract		Axial FC		Radial FC		Mean FC		FA_FCT		WM tract description
		β_{male}	β_{female}	β_{male}	β_{female}	β_{male}	β_{female}	β_{male}	β_{female}	
**MCBP**	-	0.0111	0.0094	0.0057	0.0059	0.0071	0.0073	0.0021	0.0013	middle cerebellar peduncle
**PCT**	-	-0.0053	-0.0091	-0.0140	**-0.0179**	-0.0125	**-0.0168**	**0.0209**	**0.0189**	pontine crossing tract
**GCC**	-	-0.0041	**-0.0124**	0.0007	-0.0043	-0.0003	-0.0067	-0.0029	-0.0049	genu of corpus callosum
**BCC**	-	**0.0165**	0.0076	0.0142	-0.0029	**0.0154**	-0.0007	**-0.0144**	0.0059	body of corpus callosum
**SCC**	-	0.0015	**0.0105**	0.0093	**0.0232**	0.0080	**0.0207**	**-0.0170**	**-0.0318**	splenium of corpus callosum
**FX**	-	0.0012	-0.0072	-0.0176	**-0.0207**	-0.0138	**-0.0191**	0.0201	**0.0197**	fornix
**CST**	R	0.0152	0.0055	-0.0016	-0.0005	0.0023	0.0008	0.0150	0.0031	corticospinal tract
	L	0.0040	**0.0172**	-0.0132	0.0052	-0.0098	0.0080	**0.0223**	0.0027	
**ML**	R	-0.0030	**-0.0189**	-0.0050	**-0.0228**	-0.0045	**-0.0224**	0.0039	**0.0227**	medial lemniscus
	L	-0.0065	-0.0076	-0.0058	**-0.0173**	-0.0059	-0.0154	0.0058	**0.0242**	
**ICBP**	R	0.0097	0.0006	0.0146	-0.0019	0.0140	-0.0013	**-0.0135**	0.0015	inferior cerebellar peduncle
	L	0.0109	0.0102	0.0131	0.0069	0.0132	0.0078	-0.0097	-0.0056	
**SCBP**	R	0.0004	**-0.0161**	-0.0075	**-0.0299**	-0.0057	**-0.0279**	0.0115	**0.0305**	superior cerebellar peduncle
	L	-0.0056	**-0.0194**	-0.0126	**-0.0284**	-0.0112	**-0.0276**	**0.0164**	**0.0287**	
**CP**	R	**0.0274**	**0.0240**	0.0016	**0.0146**	0.0077	**0.0173**	0.0132	-0.0012	cerebral peduncle
	L	**0.0269**	**0.0259**	0.0020	0.0122	0.0075	**0.0161**	0.0124	0.0022	
**ALIC**	R	-0.0049	**-0.0181**	-0.0123	**-0.0180**	-0.0113	**-0.0187**	0.0133	**0.0139**	anterior limb of internal capsule
	L	**-0.0142**	**-0.0133**	-0.0085	**-0.0156**	-0.0102	**-0.0154**	0.0038	**0.0142**	
**PLIC**	R	-0.0055	-0.0042	**-0.0147**	-0.0080	**-0.0133**	-0.0072	**0.0132**	0.0084	posterior limb of internal capsule
	L	-0.0080	-0.0033	**-0.0152**	-0.0092	**-0.0142**	-0.0078	**0.0147**	**0.0117**	
**RLIC**	R	0.0001	0.0044	0.0064	0.0107	0.0053	0.0098	**-0.0119**	**-0.0149**	retrolenticular part of the internal capsule
	L	-0.0047	0.0061	0.0006	0.0085	-0.0007	0.0084	-0.0090	-0.0083	
**ACR**	R	-0.0077	**-0.0125**	-0.0004	0.0000	-0.0023	-0.0030	-0.0048	-0.0074	anterior corona radiata
	L	**-0.0154**	**-0.0132**	-0.0022	-0.0011	-0.0055	-0.0039	-0.0050	-0.0031	
**SCR**	R	-0.0046	0.0013	0.0040	0.0083	0.0020	0.0070	-0.0071	-0.0081	superior corona radiata
	L	-0.0027	-0.0036	0.0022	0.0069	0.0011	0.0048	-0.0015	-0.0079	
**PCR**	R	0.0026	0.0067	**0.0139**	**0.0203**	0.0118	**0.0180**	**-0.0167**	**-0.0224**	posterior corona radiata
	L	0.0043	0.0014	**0.0141**	**0.0118**	0.0124	0.0097	**-0.0124**	**-0.0149**	
**PTR**	R	-0.0024	0.0038	0.0059	**0.0211**	0.0041	**0.0178**	-0.0107	**-0.0332**	posterior thalamic radiation
	L	-0.0004	0.0060	**0.0170**	**0.0292**	**0.0140**	**0.0249**	**-0.0227**	**-0.0410**	
**SS**	R	-0.0033	-0.0074	0.0099	-0.0013	0.0069	-0.0028	**-0.0150**	-0.0025	sagittal stratum
	L	-0.0086	-0.0099	0.0072	0.0020	0.0035	-0.0006	-0.0132	-0.0060	
**CGG**	R	-0.0094	-0.0047	-0.0043	-0.0033	-0.0056	-0.0038	-0.0059	0.0026	cingulum in the cingulate gyrus
	L	-0.0095	-0.0057	-0.0004	-0.0045	-0.0024	-0.0049	-0.0078	0.0017	
**CGH**	R	0.0146	0.0087	0.0037	0.0034	0.0066	0.0048	0.0126	0.0070	cingulum in the hippocampus
	L	0.0078	**0.0175**	0.0049	0.0110	0.0055	**0.0130**	-0.0026	-0.0002	
**FXC**	R	-0.0010	0.0092	0.0021	0.0044	0.0017	0.0058	-0.0006	0.0004	fornix (cres)
	L	0.0034	0.0021	-0.0036	0.0007	-0.0022	0.0011	0.0050	0.0007	
**SLF**	R	0.0007	**0.0109**	0.0038	**0.0141**	0.0029	**0.0139**	-0.0062	**-0.0156**	superior longitudinal fasciculus
	L	-0.0031	**0.0095**	0.0033	**0.0137**	0.0017	**0.0131**	-0.0083	**-0.0131**	
**SFO**	R	-0.0079	**-0.0150**	-0.0014	**-0.0128**	-0.0031	**-0.0141**	0.0030	**0.0121**	superior fronto-occipital fasciculus
	L	**-0.0173**	**-0.0167**	-0.0031	**-0.0118**	-0.0064	**-0.0135**	-0.0007	0.0104	
**UF**	R	0.0052	0.0132	-0.0116	0.0044	-0.0076	0.0065	**0.0249**	0.0027	uncinate fasciculus
	L	0.0055	0.0145	-0.0060	-0.0035	-0.0034	0.0009	0.0092	**0.0169**	
**TAP**	R	0.0063	-0.0064	0.0151	0.0084	0.0138	0.0053	**-0.0238**	**-0.0218**	tapetum
	L	-0.0067	-0.0025	0.0048	0.0040	0.0027	0.0028	-0.0122	**-0.0149**	

Table S3. Summary of FDR corrected p-values (q) of corresponding associations related to the interaction term between age and sex respectively. The q-values that are less than 0.05 is marked bold (no value is marked bold).

**WM tract**		**Axial FC**	**Radial FC**	**Mean FC**	**FA_FCT**	**WM tract description**
		$q_{age*sex}$	$q_{age*sex}$	$q_{age*sex}$	$q_{age*sex}$	
**MCBP**	-	0.9577	0.9845	0.9852	0.9244	middle cerebellar peduncle
**PCT**	-	0.9577	0.9845	0.9852	0.9539	pontine crossing tract
**GCC**	-	0.9555	0.9845	0.9852	0.9244	genu of corpus callosum
**BCC**	-	0.9555	0.8297	0.9176	0.7333	body of corpus callosum
**SCC**	-	0.9555	0.8297	0.9176	0.7333	splenium of corpus callosum
**FX**	-	0.9555	0.9173	0.9401	0.9244	fornix
**CST**	R	0.9555	0.9845	0.9852	0.8479	corticospinal tract
	L	0.9555	0.8297	0.9176	0.7333	
**ML**	R	0.9555	0.8297	0.9176	0.7333	medial lemniscus
	L	0.9577	0.8297	0.9176	0.7333	
**ICBP**	R	0.9555	0.8297	0.9176	0.7333	inferior cerebellar peduncle
	L	0.9577	0.8297	0.9176	0.9237	
**SCBP**	R	0.9555	0.8297	0.9176	0.7810	superior cerebellar peduncle
	L	0.9555	0.8297	0.9176	0.9237	
**CP**	R	0.9555	0.8297	0.9607	0.9237	cerebral peduncle
	L	0.9555	0.8297	0.9476	0.9237	
**ALIC**	R	0.9555	0.9173	0.9176	0.9539	anterior limb of internal capsule
	L	0.9577	0.8297	0.9476	0.7810	
**PLIC**	R	0.9577	0.8297	0.9176	0.9244	posterior limb of internal capsule
	L	0.9555	0.8297	0.9176	0.9432	
**RLIC**	R	0.9555	0.8833	0.9401	0.9275	retrolenticular part of the internal capsule
	L	0.9555	0.8297	0.9176	0.9539	
**ACR**	R	0.9555	0.9845	0.9852	0.9908	anterior corona radiata
	L	0.9555	0.9845	0.9852	0.9432	
**SCR**	R	0.9555	0.9845	0.9852	0.9791	superior corona radiata
	L	0.9555	0.9845	0.9900	0.9244	
**PCR**	R	0.9577	0.9845	0.9852	0.9432	posterior corona radiata
	L	0.9555	0.9845	0.9852	0.9432	
**PTR**	R	0.9555	0.8297	0.9176	0.7333	posterior thalamic radiation
	L	0.9555	0.8297	0.9176	0.7333	
**SS**	R	0.9555	0.8297	0.9176	0.7333	sagittal stratum
	L	0.9555	0.9845	0.9607	0.9432	
**CGG**	R	0.9555	0.9845	0.9852	0.7333	cingulum in the cingulate gyrus
	L	0.9555	0.9845	0.9852	0.9237	
**CGH**	R	0.9577	0.9845	0.9852	0.9244	cingulum in the hippocampus
	L	0.9555	0.8297	0.9176	0.9894	
**FXC**	R	0.9555	0.9845	0.9852	0.9589	fornix (cres)
	L	0.9555	0.8297	0.9176	0.9244	
**SLF**	R	0.9555	0.8297	0.9176	0.9244	superior longitudinal fasciculus
	L	0.9555	0.8297	0.9176	0.9432	
**SFO**	R	0.9555	0.8297	0.9176	0.7810	superior fronto-occipital fasciculus
	L	0.9577	0.8297	0.9176	0.8419	
**UF**	R	0.9555	0.8297	0.9176	0.7333	uncinate fasciculus
	L	0.9577	0.9845	0.9852	0.9244	
**TAP**	R	0.9555	0.9845	0.9852	0.9908	tapetum
	L	0.9555	0.9845	0.9852	0.9244	
